# DBSolve Optimum: a software package for kinetic modeling which allows dynamic visualization of simulation results

**DOI:** 10.1186/1752-0509-4-109

**Published:** 2010-08-10

**Authors:** Nail M Gizzatkulov, Igor I Goryanin, Eugeny A Metelkin, Ekaterina A Mogilevskaya, Kirill V Peskov, Oleg V Demin

**Affiliations:** 1 Institute for Systems Biology SPb, Sankt-Peterburgh, Russia; 2 University of Edinburgh, Edinburgh, UK; 3 A.N. Belozersky Institute of Physico-Chemical Biology of Moscow State University, Moscow, Russia

## Abstract

**Background:**

Systems biology research and applications require creation, validation, extensive usage of mathematical models and visualization of simulation results by end-users. Our goal is to develop novel method for visualization of simulation results and implement it in simulation software package equipped with the sophisticated mathematical and computational techniques for model development, verification and parameter fitting.

**Results:**

We present mathematical simulation workbench DBSolve Optimum which is significantly improved and extended successor of well known simulation software DBSolve5. Concept of "dynamic visualization" of simulation results has been developed and implemented in DBSolve Optimum. In framework of the concept graphical objects representing metabolite concentrations and reactions change their volume and shape in accordance to simulation results. This technique is applied to visualize both kinetic response of the model and dependence of its steady state on parameter. The use of the dynamic visualization is illustrated with kinetic model of the Krebs cycle.

**Conclusion:**

DBSolve Optimum is a user friendly simulation software package that enables to simplify the construction, verification, analysis and visualization of kinetic models. Dynamic visualization tool implemented in the software allows user to animate simulation results and, thereby, present them in more comprehensible mode. DBSolve Optimum and built-in dynamic visualization module is free for both academic and commercial use. It can be downloaded directly from http://www.insysbio.ru.

## Background

Kinetic modeling is one of the main tools of computational systems biology aimed at quantitative description of intracellular dynamic processes. The term kinetic model refers to a system of algebra-, integral- or delay- differential equations that determine the temporal and steady state of the corresponding biological system. The model of a particular system of biochemical reactions is usually represented by a system of mechanistic differential equations and includes multiple parameters which values must be estimated on the basis of experimental data. There are, at least, three stages in study of a biochemical system by means of kinetic modeling [1]. The first stage consists of constructing kinetic model of the biochemical system on the basis of all available information. The second stage is numerical solution of the resulted system of (differential) equations and the analysis of the results of simulations. The third stage allows modeler to test predictive power of the kinetic model by comparison of results of calculation with experimental data, and also to generate a number of hypotheses about dynamic and regulatory properties of the biological processes under study. Each of the stages requires specialized software facilitating model handling. Moreover, to construct kinetic models, analyze simulation results and compare them with experimental data, specialized visualization tools should be available. There are two types of visualization tools available in various simulation software packages. First one provides visualization of a biochemical system corresponding to model developed. The biochemical system is usually visualized as static map representing metabolites interconnected with multiple reactions (see [2], for example). Second type of the tools provides visualization of model simulation results. They are usually visualized as plot with single or multiple curves representing time series or dependences of metabolite concentrations and fluxes on a parameter at steady state (see [3], for example). However, to represent dynamic behavior of the system as a whole it would be very useful to transfer model simulation results to static scheme of corresponding biochemical system and animate changes in metabolite concentrations and fluxes. The first step in this direction has been done in SimWiz visualization software [4] which allows to animate concentrations of metabolic system. However, this software fails (i) to animate changes in reaction rates (fluxes), (ii) to display scale of animated metabolite concentrations and intrinsic animation time or independent parameter value, (iii) to save animation as video file, (iv) to edit and annotate of animated visual map. To fill the gap we introduce a concept of dynamic visualization of simulation results enabling user to display simulation results via animation of static scheme of biochemical system. In this paper we present DBSolve Optimum which is software designed to develop, analyze kinetic models and visualize simulation results and focus on implementation of the concept of dynamic visualization in DBSolve Optimum as built-in dynamic visualization module.

## Implementation

DBSolve Optimum is significantly improved and extended successor of well known simulation software DBSolve5 [5]. Program has been written using C++ language and compiled with Borland Builder C++ compiler. DBSolve Optimum framework consists of two parts: DBSolve Optimum Developer Environment (DODE) и DBSolve Optimum Player (DOP). DODE is designed for creation, editing, simulation, analyzing and visualization of kinetic models. DOP is focused on animation of static visual map of the kinetic model on the basis of simulation results (time series and dependence of steady state on parameter).

**Figure 1:**
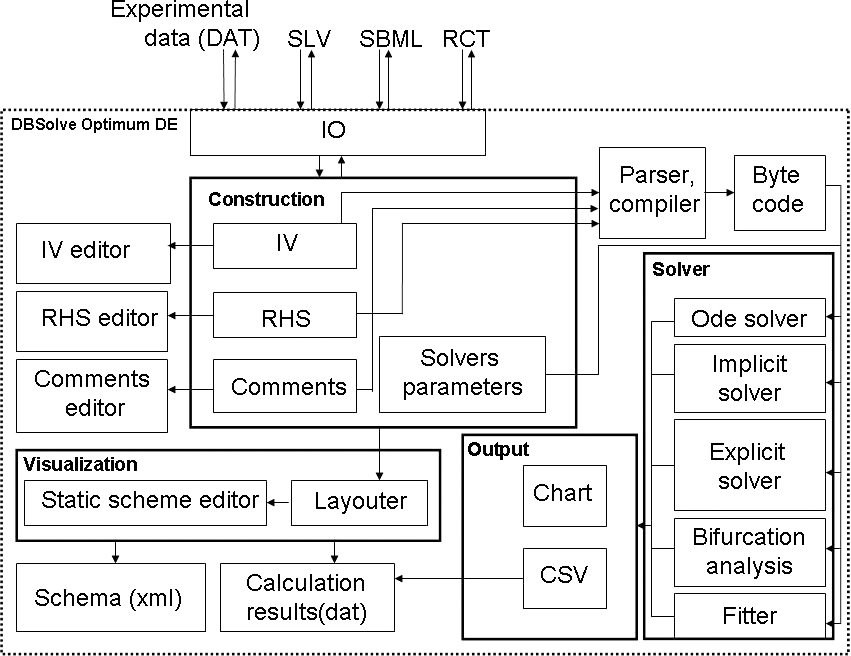
**Architecture of the DBSolve Optimum Developer Environment**. Abbreviations: IO - Input/Output module, DAT, SLV, SBML, RCT - data file formats, IV - Initial values, RHS - Right hand sides, CSV - text file of CSV format.

**Figure 2:**
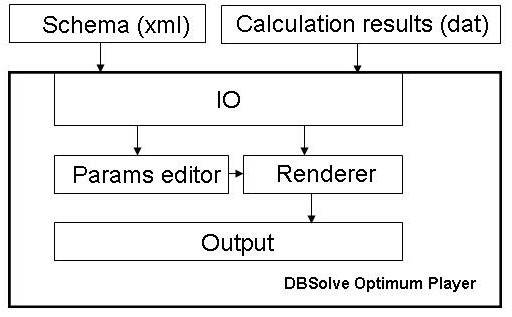
**Architecture of the DBSolve Optimum Player**. Abbreviations: IO - Input/Output module, Params editor - Parameters editor.

Figs 1 and 2 represent architecture of DODE and DOP, correspondently. DODE workflow (Fig. 1) consists of data input (IO module), creation and editing model (Model Construction module), its verification and analysis (Solver module) and output of simulation results (Output module) and their visualization (Visualization module). Work with DODE starts with creation of new model or download existing file in "SLV" or "SBML" format. "SLV" (SoLVe) is internal format of DBSolve Optimum which includes both detail information about kinetic model and description of user settings. "SBML" (Systems Biology Markup Language) format [6] is designed for storage of kinetic models and their exchange between modelers which use various software packages for model development and analysis. DODE supports SBML version L2V4 (Level 2 Version 4) and uses libSBML library for Import/Export of SBML files.

### Model construction module of DODE

Development of kinetic model starts with construction of the stoichiometric matrix of corresponding biochemical system. DODE enables user either to enter the matrix directly or download it via "RCT" (ReaCTions) file. This is ASCII (American Standard Code for Information Interchange) text file with delimiters. Each string of this file describes stoichiometry of a reaction of the kinetic model. RCT format have been realized to simplify input of stoichiometric matrixes. The description of this format is given in additional file 1: supplementary materials. On the basis of the stoichiometric matrix DODE creates template kinetic model which reaction rates are described in accordance with mass action law. ODE (Ordinary Differential Equation) system and values of model parameters and variables are presented in the form of two plain-text files. Right hand sides of ODE system including reaction rates, conservation laws and explicit functions are defined in "RHS" (Right Hand Side) file. Initial values of all variables of the model and values of all other parameters are defined in "IV" (Initial Values) file. Each line of the "IV" and "RHS" files has the following syntax: "variable = expression;". Each line of the "RHS" and "IV" should be separated and ended with a comma point delimiter. In comparison to the previous version (DBSolve 5) DBSolve Optimum allows to use conditional operators such as: if (condition) {operators} else {operators}. This possibility allows to simulate piecewise continuous functions into the right hand sides of the differential equations. At the next stage of model construction user can change the rate equations and values of parameters manually by means of "IV editor", "RHS editor" to take into account all peculiarities of the dynamics and regulations of the biochemical system.

### Model analysis and calculation modules of DODE

Analysis of the kinetic model, its fitting to experimental data and simulation of verified model is accomplished by means of following calculation modules: "Ode solver", "Implicit solver", "Explicit solver", "Bifurcation analysis", "Fitter". Operation of each calculation module is specified by a set of corresponding control parameters (see user manual for details). Before running calculation DODE parses and validates "RHS" and "IV" texts entered by user and produces byte code. This code is further used to calculate right-hand sides of the ODE system corresponding to the model. Parser program code of DODE has been generated using Flex and Bison tools (free lexical and grammar generators available at [7] and [8]). Below we briefly describe the calculation modules of DODE.

#### ODE Solver

To describe dynamics of the kinetic models DBSolve Optimum uses a popular LSODE (Livermore Solver for Ordinary Differential Equations) algorithm [9]. These methods have special subroutines for getting output for user-defined time points, which is essential for fitting algorithms. In additional file 1: supplementary materials in section C an example of calculation of time dependence of NADH (Nicotinamide Adenine Dinucleotide (reduced form)) production by α-ketoglutarate dehydrogenase reaction is presented.

#### Explicit Solver

Users may have their own particular equation which they require solving and wish to be applied to a set of experimental data. DBSolve Optimum offers the facility to encode and solve such "explicitly stated" formulae. They should be typed at the bottom of the RHS window in the section "Explicit Function" and then solved by turning to the "Explicit Solver" tabbed page, where the appropriate variable, interval of variation of parameters and an initial step can be entered. In additional file 1: supplementary materials in section C an example of calculation of Succinate thiokinase initial rate dependence on the concentration of its substrates and products is presented.

#### Implicit Solver

This method allows the user to trace the changes in the steady state of the system as a result of variation of one or more of its parameters. This procedure is very useful for determining any functional dependencies (such as overall steady-state flux, control coefficients, product concentration, some parameters of the model) against any external (substrate concentrations) or internal (enzyme concentrations) or some model parameter. It is especially useful in the case of non-linear algebraic systems which have no explicit solution or have multiple or unstable solutions. DBSolve Optimum includes a general continuation procedure, based on a tangent predictor continuation scheme [10]. A modified Newton corrector is employed which makes adaptive step sizes on the basis of estimates from the current tangent and secant vectors. This minimizes the possibility of jumping from one branch of a curve to another, and allows the user to optimize the next step size according to computed points on the curve. In DBSolve Optimum 3D plot feature for "Implicit solver" has been realized. In additional file 1: supplementary materials in section C dependence of stationary glutamate consumption rate on concentration of external glutamate calculated from kinetic model of mitochondrial Krebs cycle is presented.

#### Bifurcation Analysis

Bifurcation theory is a more systematic and general theory of non-linear systems than the standard, steady-state analysis of metabolic networks. Computation of one or two-parameter bifurcation diagrams can quickly inform the user about what is possible for, or prohibited by, a particular type of non-linear model [11]. To calculate one and two-parameter diagrams of Equilibrium, Fold, Hopf, Flip and Focus-node bifurcations DBSolve Optimum uses numerical methods similar to LOCBIF (LOCal BIFurcation) [10]. All algorithms have been rewritten in "C ++" and modified to integrate with the DBSolve Optimum object-oriented environment. The Bifurcation Analyzer uses the same numerical continuation code as the Implicit Solver, but it is expanded with routines for the evaluation of bifurcation functions and calculating eigenvectors. Bifurcations have been found at points where black rectangles are drawn on the plot. Example of application of the solver can be found in [1].

#### Fitter

This method can be used to fit a model to experimental data (thereby discovering the values with appropriate error margins of the models parameters under the conditions of the experiment).

The fitting/optimization can exploit either a zero-order [12] or first-order [13,14] algorithm. Fitting procedure often encounters difficulties caused by multiple minima, which may be a particular problem when many parameters are fitted. The "best" fit might not be easily found; however, to check the quality of the procedure, the standard deviation and confidence intervals for every active parameter as well as an ANOVA (ANalysis Of VAriance) table are shown in the "Message window" to help users make their assessment. When fitting to experimental data, the objective residual function between theoretical and experimental points is calculated according to a least square or absolute value (modulus) formula. These are defined by the following equations:

$$\text{F}0=\sum {\left(\text{Yti}-\text{Yei}\right)}^{\text{2}}$$

 F0=∑|Yti−Yei|

$$\;\text{F}0=\sum {\left(\text{Yti}-\text{Yei}\right)}^{\text{2}}/{\text{Yei}}^{\text{2}}$$

 F0=∑|Yti−Yei|/|Yei|

where Yti and Yei are the theoretical and experimental values, respectively and F0 is discrepancy.

In additional file 1: supplementary materials in section C examples of fitting of experimental data by means of ODE Solver, Explicit Solver and Implicit Solver are presented.

### Model visualization modules

Simulation results and schematic representation of biochemical system of the model can be visualized by DBSolve Optimum in two possible ways.

#### Conventional visualization of modeling results and its implementation in DODE

Each calculation module can either save calculation results as text file of CSV (Comma Separated Value) format or plot them as a graph. The second option has been implemented in DODE on the basis of TeeChart package [15]. In comparison to DBSolve5, 3D plot feature for "ODE solver" and "Implicit solver" has been implemented in DODE. This feature allows user to create a plain text file with three columns (see more detail description in additional file 1: supplementary materials): values of time (first column), values of the parameter (second column) and values of variable. This file can be used as input for any program (for example Excel, TechPlot) to make a 3D chart.

#### Dynamic visualization of model simulation results and its implementation in DBSolve Optimum Framework

**Figure 3:**
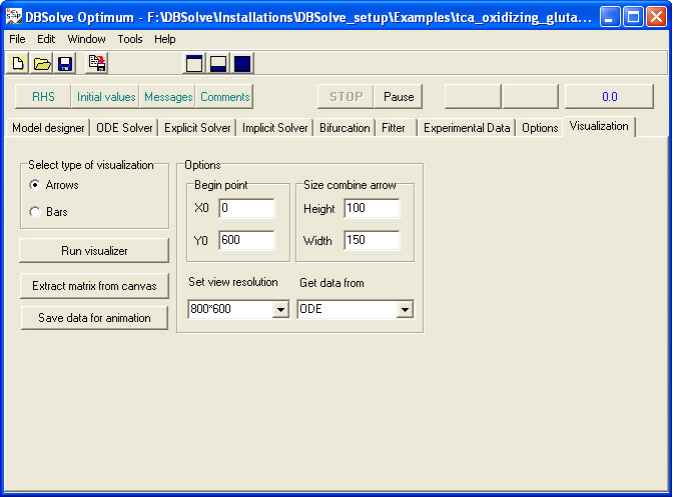
**DODE main window with opened Visualization tab**.

**Figure 4:**
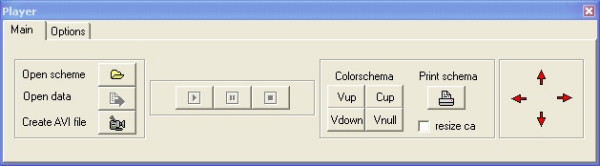
**DBSolve Optimum Player interface**.

To understand behavior of the biological system as a whole we have developed special visualization technique allowing to animate simulation results (Dynamic Visualization) and implemented it in DBsolve Optimum. The main idea of the technique consists of (1) construction of visual map of the biological system (and corresponding model) and (2) animation of the visual map, i.e. reproducing dynamic changes in concentrations and fluxes by altering shapes and volumes of geometrical objects corresponding to these model variables. Graphical objects corresponding to biological entities and reactions of the model can change their volume and shape in accordance to the calculated values of corresponding concentrations and fluxes. Dynamic Visualization (animation) can be used to represent both kinetic (time dependent) response of the model to any perturbations and dependences of steady state concentrations and fluxes to any model parameters. This Dynamic Visualization technique has been implemented in DBSolve Optimum Framework in following manner. Initial construction of the static visual map (pathway) for kinetic model and generation of data file with simulation results has been implemented in DODE as Dynamic Visualization Module (DVM) (see Fig. 3 for interface of the DVM). Animation of the visual map on the basis of the simulation results has been implemented in DOP (see Fig. 4 for DOP interface). Architecture of DVM is shown in Fig. 1 (see Visualization unit) and architecture of DOP is presented by Fig. 2. Both DVM and DOP are based on OpenGL library [16] to draw graphical objects. DVM uses stoichiometric matrix of the model as input data to construct initial visual map (see Layouter module in Fig.1). At the next step user can edit and annotate the initial visual map. For example, user can draw the arrows and nodes ("Arrow animation" mode) or bars (in "Bar animation" mode) to arrange the graphic objects in desirable manner.

Output of the DVM is visual pathway map (as XML file) and calculation results produced by DODE solvers and saved as special plain text file with "PLT" (PLain Text) extension (see visualization block on Fig.1). XML (eXtensible Markup Language) file is designed to store geometry of basic graphic objects. The XSD (XML Schema Definition Language) schema of this XML file can be found on our website [17]. The PLT file is designed to store simulation results produced by ODE and/or Implicit Solvers (see below for detailed description of the DBSolve Optimum functionalities). PLT file is an ASCII delimited text file. First row of the PLT file specifies the number of metabolites of the kinetic model - N1. Second row of the file specifies number of reactions of the kinetic model - N2. Third row of the file specifies titles of the data columns written below. For example, "X[0]" - time, "Metabolite1" - concentration of metabolite1, ...., "MetaboliteN1" - concentration of metaboliteN1, "Reaction1" - flux through reaction1, ..., "ReactionN2" - - flux through reaction1. First data column of the PLT file specifies time steps t_i (in case of usage of ODE Solver as generator of numerical data) or steps in parameter change, p_i (in case of usage of Implicit Solver as generator of numerical data). Next N1 columns of the PLT file specify changes in concentrations and next N2 columns specify changes in fluxes.

Static scheme of the biochemical system (visual map saved as XML file) and simulation results (saved as PLT file) are input data for DOP. On the basis of the input data DOP is able to animate visual map and save the animation as video file. Indeed, to reproduce the animation corresponding to the selected model, the user should download in DOP the XML file with the constructed visual map and corresponding PLT file generated in DVM (see Fig. 2). Before starting with playback of the animation user should set the values of parameters of DOP (see Params editor box on Fig. 2) responsible for the visual properties of the animation.

**Figure 5:**
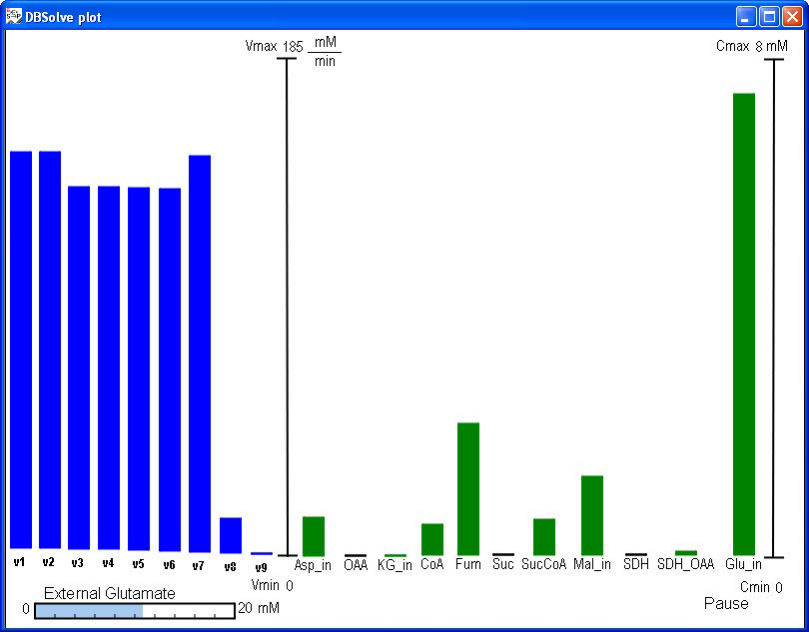
**Different modes of the dynamic visualization: "Bar animation" mode**. An example of animation: the dependence of steady state concentrations and fluxes of the Krebs cycle on external glutamate concentration. Blue bars correspond to the values of the reaction rates and green bars correspond to the metabolite concentration values.

**Figure 6:**
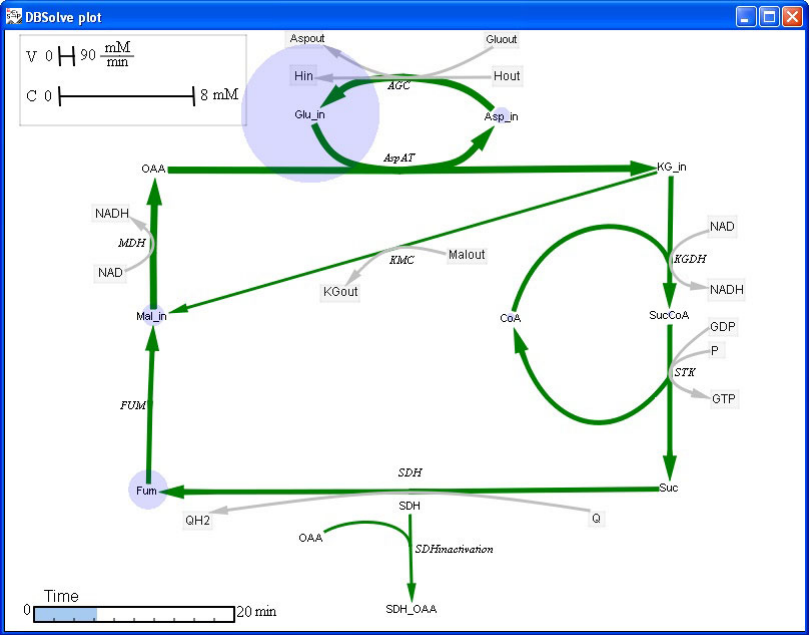
**Different modes of the dynamic visualization: "Arrow animation" mode**. An example of visualization: time response of the concentrations and fluxes of the Krebs cycle resulted from changes in external glutamate concentration from 2 mM to 10 mM. **Changes in width of arrows **and circles radius **visualize changes in reaction rates **and metabolites concentrations correspondingly.

DBSolve Optimum allows visualization of simulation results in two different ways: "Bar animation" and "Arrow animation" (see corresponding options for construction of visual map in DVM interface Fig. 3). "Bar animation" mode (Fig. 5) allows modeler to create a set of the bars corresponding to reactions and entities of the system. The height of the rectangles corresponds to the value of either reaction rate or concentration. Animation in this mode implemented in DOP as changes in heights of the bars in accordance to data provided by simulations. "Arrow animation" mode (Fig. 6) allows us to create static map consisting of nodes (biological entities) and the directed edges (reactions). Variation of concentration of i-th metabolite, *C_i_*, is represented by change in circle radius, *r_i_*, around the corresponding node in such a way that the concentration is directly proportional to circle radius:

$$r_{i}=\left\{ \begin{array}{l} 0,\;C_{i}<{\bar{C}}_{\min}\\ \frac{C_{i}-{\bar{C}}_{\min}}{{\bar{C}}_{\max}-{\bar{C}}_{\min}}\cdot r_{\max},\;\\ {\bar{C}}_{\min}\leq C_{i}\leq {\bar{C}}_{\max}\\ r_{\max},\;C_{i}>{\bar{C}}_{\max} \end{array}\right.$$

where ${\bar{C}}_{\min}$, ${\bar{C}}_{\max}$ are values of minimal and maximal concentration and *r*_max _is maximal radius of the circles specified by the user. There are two options to visualize changes in reaction rates. First one visualizes the value of the reaction rate, *V*_*i*_, as the thickness of the arrow, *h*_*i*_, connecting substrates and products of the reaction (Fig. 6), thus the thickness of an arrow is directly proportional to the value of the reaction rate:

$$h_{i}=\left\{ \begin{array}{l} 0,\;V_{i}<{\bar{V}}_{\min}\\ \frac{V_{i}-{\bar{V}}_{\min}}{{\bar{V}}_{\max}-{\bar{V}}_{\min}}\cdot h_{\max},\\ \;{\bar{V}}_{\min}\leq V_{i}\leq {\bar{V}}_{\max}\\ h_{\max},\;V_{i}>{\bar{V}}_{\max} \end{array}\right.$$

Where ${\bar{V}}_{\min}$, ${\bar{V}}_{\max}$ are values of minimal and maximal fluxes, *h*_max _is maximal thickness of the arrows specified by the user. The second one represents the value of the reaction rate as a circle, thus the circle radius is directly proportional to the reaction rate.

Further, clicking button "Play" allows viewing animation and clicking "Save to avi" saves the animation in AVI format.

In additional file 1: supplementary materials we present in details all functionalities of DBsolve Optimum and exemplify them in kinetic model of mitochondrial Krebs cycle.

## Results

There are many stand-alone software packages available for systems biology and kinetic modeling [18]. Most are available as a result of the efforts of the SBML community [6]. However, only a few contain a full range of tools to allow kinetic model creation, parameter fitting and analysis. DBSolve Optimum is one of these packages. DBSolve is a software environment for creation, analysis and visualization of kinetic models of biological processes. Development of DBSolve as a package for kinetic modeling is tightly coupled with development of group of kinetic modeling of Institute for Systems Biology SPb [19]. Evolution of DBSolve is caused by the biomedical and biotechnological problems which have been addressed by the group for more than 10 years. A number of versions have been released during more than 10 years of software development [5,20-22]. During this period DBSolve has been extensively used in Institute for Systems Biology SPb [23,24], Thomas Jefferson University [25,26], Edinburgh University [27,28], Moscow State University [29,30] and by GlaxoSmithKline [31-33] to create hundreds of kinetic models for both research and teaching. The package has built-in algorithms and tools for constructing models and fitting parameters to the experimental data. All the models are considered to be systems of non-linear ordinary differential equations and/or non linear algebraic differential equations with arbitrary right hand sides. These features allow modelers to expand the class of possible applications to include chemical, PK/PD [34], ecological or other biomedical systems [35].

DBSolve includes the following methods:

1. Generation of stoichiometric matrix based on the list of the reactions describing the system;

2. Automatic analysis of the stoichiometric matrix;

3. Automatic generation of the systems of ordinary differential equations and conservation laws based on the stoichiometric matrix;

4. Calculation of functional dependencies defined explicitly;

5. Numerical solution of non-linear ODE system and visualization of the solution;

6. Calculation of functional dependencies defined implicitly as a system of algebraic equations (generally nonlinear);

7. Automatic search of optimal values of the parameters of a system based on the experimental data (fitting);

8. Analysis of stability of the dynamic system (bifurcation) and calculation of the control coefficients as defined in metabolic control analysis.

### Comparison of DBSolve Optimum with other packages for kinetic modeling

DBSolve Optimum belongs to the family of software packages designed for creation, analysis and simulation of kinetic models of biological systems. More than 50 different programs able to assist in quantitative description and visualization of biological systems are presented in web site [36]. These programs vary in functionality, availability, tasks to be solved and other characteristics. Indeed, all the software packages can be classified into following groups: (i) programs designed for opening and editing sbml files, (ii) programs for model annotation and visualization of metabolic maps, (iii) programs focused on comprehensive work with kinetic model of a biological system including model development, verification, analysis and visualization. Performance of DBSolve Optimum is exemplified in details in additional file 1: supplementary materials and many other publications [37,38]. DBSolve Optimum belongs to the last class and we will compare its functionality to the similar (comprehensive) packages presented in [36].

As follows from the comparison (Table 1) DBSolve Optimum includes the most comprehensive set of tools required for development of kinetic model of biochemical system, its analysis and verification against experimental data and visualization of simulation results. For example, only DBSolve Optimum has a tool for calculation and plotting of bifurcation diagrams, simulation of explicitly defined function and fitting the explicit function to experimental data. Similar to Copasi DBSolve Optimum is able to perform fitting the model to experimentally measured time dependence and dependence of steady state on parameter simultaneously [39]. But in addition DBSolve Optimum can simultaneously fit the model to experimental data describing both time dependence (Fitting ODE), dependence of steady state on parameter (Fitting Implicit) and dependence of explicitly defined function on parameter or variable of the model (Fitting Explicit). At the same time DBSolve Optimum does not include some features which are presented by other software packages, like Stochastic ODE Solver.

**Table 1:** Comparison of DBSolve Optimum functionality with that of DBSolve5, Copasi, Teranode, Cell Designer and SBML Pet

	DBSolve Optimum	DBSolve5	Copasi	TERANODE suite	Cell Designer	SBML PET
**Create model**	+	+	+	+	+	-
**SBML support**	+	-	+	+	+	+
**ODE Solver**	+	+	+	+	+	+
**Stochastic ODE Solver**	-	-	+	-	-	-
**Steady state analysis**	+	+	+	-	-	-
**Explicit Solver**	+	+	-	-	-	-
**Implicit Solver**	+	+	+	-	-	-
**Fitting ODE**	+	+	+	+	+	+
**Fitting Explicit**	+	+	-	-	-	-
**Fitting Implicit**	+	+	+	-	-	-
**Bifurcation analysis**	+	+	-	-	-	-
**Plotting of calculation results 2D/3D**	+/+	+/-	+/-	+/-	+/-	- /-
**Animation of calculation results**	+	-	+	-	-	-
**Commercial**	-	-	-	+	-	-
**Multiplatform**	-	-	+	+	+	+

A list of tools implemented (marked by "+") or not implemented (marked by "-") in corresponding software package is presented.

### Visualization with DBSolve Optimum

DBSolve Optimum Visualization Module allows user (1) to create visual map using stoichiometric matrix, (2) to create stoichiometric matrix from visual map, (3) to export of the visual map to raster or vector graphical format files and (4) to animate visual map. Automatic construction of the basis of the visual map (clause 1) allows modeler to facilitate substantially process of creation of metabolic map corresponding to the model developed. In framework of this option DBSolve Optimum creates set of graphical objects which are interconnected by reactions in accordance to stoichiometric matrix. To create the appropriate map, user should drag these objects in appropriate places on the canvas. Other option of Visualization (2) allows to solve inverse problem, namely to construct automatically the stoichiometric matrix from the visual map. The export of the visual map to raster or vector graphical format (3) allows modeler to use maps for presentations and reports. Animation of the visual map (4) is dynamic representation of *in silico *simulations.

To create Dynamic Visualization (animation) of simulation results in DODE, user should open "Visualization" tabbed page of DBSolve Optimum (see Fig. 3). Then, choose mode of visualization ("Bar animation" and "Arrow animation") ticking either "Arrows" or "Bars" in "Select type of visualization" section. Clicking "Run visualizer" button user opens visual map with graphical objects representing metabolite concentrations and reaction rates of the model. If user chooses "Bar animation", both concentrations and rates are represented by bars. If "Arrow animation" mode has been chosen, these objects are arrows for rates and cycles for the concentrations. User can edit and annotate the visual map drawing any additional arrows and nodes ("Arrow animation" mode) and bars (in "Bar animation" mode) as well as arranging the graphic objects in desirable manner. Using "Tool Bar" one can add other graphic objects (arrows, text, bars etc) to the visual map and save it as XML file. To save data, which will be used to animate objects on visual map, user should choose Solver (ODE or Implicit) for generation of simulation data in "Get data from" window of "Options" section. Then, run the model clicking "Save data for animation" and save them as PLT file.

When static visual map (XML file) and simulation results (PLT file) are generated user can download them to DOP (see Fig. 4). The basic functionalities of DOP are to animate visual map and to save results to AVI file. Examples of the Dynamic Visualization of simulation results of kinetic model of Krebs cycle [35] are presented in Figs. 5 and 6. Indeed, Fig. 5 represents "Bar animation" mode of the dependence of steady state concentrations and fluxes of the Krebs cycle on external glutamate concentration which is parameter of the model. Fig. 6 demonstrates "Arrow animation" mode of time response of the concentrations and fluxes of the Krebs cycle resulted from changes in external glutamate concentration from 2 mM to 10 mM. Kinetic model of Krebs cycle (additional file 2: kinetic model of TCA cycle), two static maps for "Bar" (additional file 3: Schematic representation of TCA cycle kinetic model in bars animation) and "Arrow" (additional file 4: Schematic representation of TCA cycle kinetic model in arrows animation) animation mode and two files with calculation results for "Bar" (additional file 5: numerical data for bars animation) and "Arrow" (additional file 6: numerical data for arrows animation) animation modes are included in distributive of DBSolve Optimum which can be downloaded from [40]. To create these XML and PLT files on the basis of file of the Krebs cycle model several consecutive steps have been performed. These steps are described in details in additional file 1: supplementary materials in section A.

Table 2 represents results of the comparison of DBSolve Optimum visualization functionality with that of other similar software packages. It is evident from the comparison DBSolve Optimum includes the most comprehensive set of tools required for visualization of simulation results. Indeed, similar to COPASI, Teranode, CellDesigner and SBML PET, DBSolve enables user to visualize simulation results using conventional tools such as plots of corresponding time series or dependences of steady state on parameter. At the same time, similar to SimWiz and COPASI, DBSolve is able to animate simulation results. But in addition DBSolve Optimum can allows user (i) to plot simulation results against experimental data, (ii) to edit and annotate visual map, (iii) to animate changes in both concentrations and reaction rates and (iv) to use 3 different types of the dynamic visualization and save animation as video file.

**Table 2:** Comparison of DBSolve Optimum visualization functionality with that of Copasi, Teranode, Cell Designer, SBML Pet and SimWIZ

Features of visualization	DBSolve Optimum	Cell Designer	COPASI (*)	Teranode	SBML PET	SimWiz	SimWiz 3D
**Create scheme of the model**	+	+	-	+	-	+	+
**Annotate scheme **	+	+	-	+	-	-	-
**Add user shapes to scheme**	+	+	-	+	-	-	-
**Add user text to scheme **	+	+	-	-	-	-	-
**Plot simulation results**	+	+	+	+	+	-	-
**Plot experimental data versus simulations result**	+	-	+	-	+	-	-
**Animate changes of metabolite concentrations on the scheme**	+	-	+	-	-	+	+
**Animate changes of reaction rates on the scheme**	+	-	-	-	-	-	-
**Number of animation types**	3	0	1	0	0	1	2
**Time or Parameter changing progress bar **	+	-	-	-	-	-	-
**Save animation to AVI**	+	-	-	-	-	-	-

A list of tools implemented (marked by "+") or not implemented (marked by "-") in corresponding software package is presented.

## Conclusions

In this article we present DBSolve Optimum. The software package has been successfully employed for dynamic modeling and visualization of microbial central metabolism and gene regulation, signal transduction pathways and mitochondrial oxidative phosphorylation. Broad functionalities of DBSolve Optimum are able to address most problems arising in systems biology. Implementation of dynamic visualization tool in DBSolve Optimum has been described in details. This tool allows user to animate simulation results and, thereby, present them in more comprehensible mode. The visualization module of DBSolve Optimum is an important feature which provides essential functionality for presentation of modeling results and communication to biologists and medics.

## Availability and Requirements

DBSolve Optimum runs on Windows platforms. Also it can be run under Linux platform using Wine: free Win32 implementation [41]. DBsolve Optimum binaries and user guide could be downloaded from [42]. DBSolve Optimum visualization module uses OpenGL library. Antialiasing option of OpenGL library is on by default. This means that user video adapter should support OpenGL acceleration. If this is not the case it works much slower. DBSolve Optimum binaries are distributed in accordance to BSD like license. Text of the license can be downloaded together with DBSolve Optimum [43].

## Abbreviations

DODE: DBSolve Optimum Developer Environment; DOP: DBSolve Optimum player; SLV: (SoLVe) is internal format of DBSolve Optimum; RCT: (ReaCTions) file; SBML: Systems Biology Markup Language; ASCII: American Standard Code for Information Interchange; ODE: Ordinary Differential Equation; RHS: Right Hand Side; IV: Initial Values; LSODE: Livermore Solver for Ordinary Differential Equations; NADH: Nicotinamide Adenine Dinucleotide (reduced form); CSV: Comma Separated Value format; DVM: Dynamic Visualization Module; PLT: PLain Text extension; XML: eXtensible Markup Language file; XSD: XML Schema Definition Language.

## Authors' contributions

IG, NG, OD designed and NG programmed the DBSolve Optimum. OD, NG, EuM wrote software documentation. KP, EuM prepared web site materials. OD, KP, EuM, EkM, NG tested DBSolve Optimum. All co-authors contributed to the conception and design of the manuscript as well as drafted and revised the manuscript. All authors read and approved the final manuscript.

## Supplementary Material

Additional file 1 **supplementary materials**. file contains supplementary materials describing in details (1) main features of DBSolve Optimum (2) kinetic model of Krebs cycle as an example of DBSolve implementation to model biochemical system. Click here for file

Additional file 2 **kinetic model of TCA cycle**. DBSolve Optimum kinetic model file. file contains kinetic model of TCA cycle in internal DBSolve language. Click here for file

Additional file 3 **Schematic representation of TCA cycle kinetic model in bars animation**. DBSolve Optimum schema file. file contains scheme of kinetic model of TCA cycle in xml format designed for bar animation. Click here for file

Additional file 4 **Schematic representation of TCA cycle kinetic model in arrows animation**. DBSolve Optimum schema file. file contains scheme of kinetic model of TCA cycle in xml format designed for arrows animation. Click here for file

Additional file 5 **numerical data for bars animation. DBSolve Optimum Player animation data**. file contains numerical data designed for bar animation of simulation results of the kinetic model of TCA cycle. Click here for file

Additional file 6 **numerical data for arrows animation**. DBSolve Optimum Player animation data. file contains numerical data designed for arrow animation of simulation results of the kinetic model of TCA cycle. Click here for file
