# Supplementary material for: DBSolve Optimum: a software package for kinetic modeling which allows dynamic visualization of simulation results
Source: BMC Syst Biol. 2010 Aug 10;4:109. doi: 10.1186/1752-0509-4-109 (PMC2925829; doi:10.1186/1752-0509-4-109)
Supplement: Additional file 1 — supplementary materials. file contains supplementary materials describing in details (1) main features of DBSolve Optimum (2) kinetic model of Krebs cycle as an example of DBSolve implementation to model biochemical system. [file 1752-0509-4-109-S1.DOC]

DBSolve Optimum: a software package for kinetic modeling which allows dynamic visualization of simulation results.

### Nail Gizzatkulov^1§^, Igor Goryanin ^2^, Eugeny Metelkin^1^, Ekaterina *Mogilevskaya*^1^, Kirill Peskov^1^, Oleg Demin^1,3^

^1^ Institute for Systems Biology SPb, Sankt-Peterburgh, Russia

^2^ University of Edinburgh, Edinburgh, United Kingdom

^3^ A.N. Belozersky Institute of Physico-Chemical Biology of Moscow State University, Moscow, Russia

^§^Corresponding author

Email addresses:

NG: [gizzatkulov@gmail.com](mailto:gizzatkulov@gmail.com)

EkM: mogilevskaya@insysbio.ru

EuM: metelkin@insysbio.ru

IG: [goryanin@inf.ed.ac.uk](mailto:goryanin@inf.ed.ac.uk)

KP: peskov@insysbio.ru

OD: demin@insysbio.ru

***Supplementary Materials:***

A. How to create and run Dynamic Visualization in DBSolve Optimum

To create Dynamic Visualization (animation) of simulation results, open “Visualization” tabbed page of DBSolve Optimum (see Fig. A1). Then, choose mode of


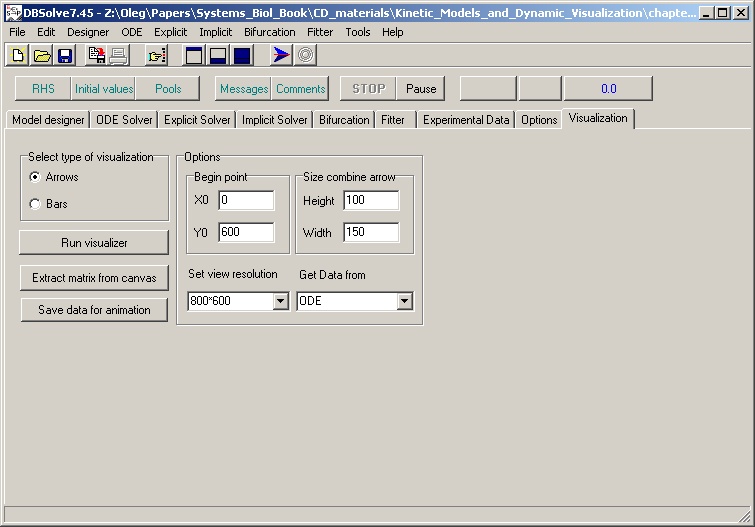


**Fig. A1.**Visualization tabbed page in DBSolve Optimum.

visualization (“Bar animation” or “Arrow animation”) ticking either “Arrows” or “Bars” in “Select type of visualization” section. Clicking “Run visualizer” button you can open visual map (see Fig. A2 for “Arrow animation” mode and Fig. A3 for “Bar animation” mode) with pre-defined layout of graphical objects corresponding to concentrations and reaction rates.


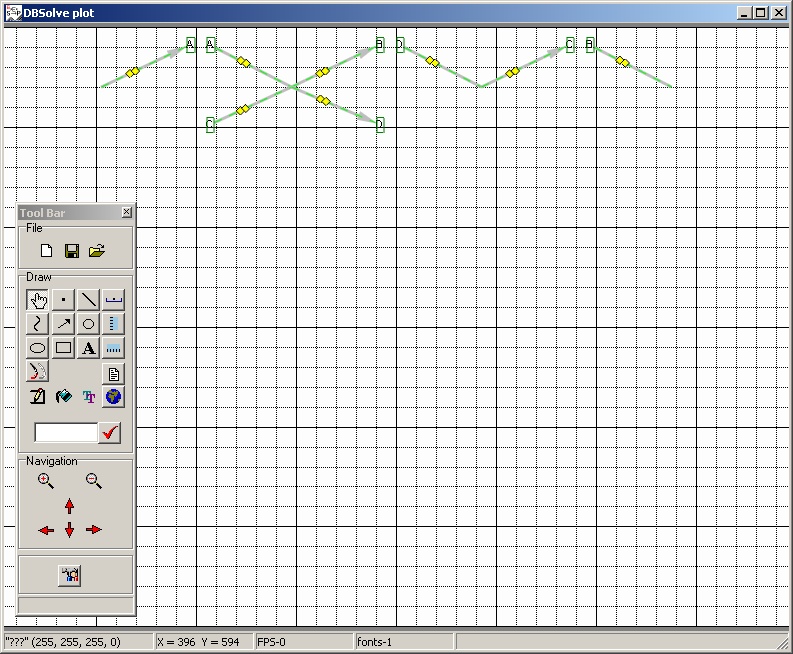


**Fig. A2.** “Arrow animation” mode.

To arrange the graphic objects in desirable manner user can move the arrows and nodes (“Arrow animation” mode) and bars (“Bar animation” mode) in the visual map, draw new graphical elements and annotate them. Using “Tool Bar” one can add other graphic objects (arrows, text, bars etc) to the visual map and save it as XML file (see detail description of the “Tool Bar” at the end of the section).

*Important Note:* When visual map is being constructed in “Arrow animation” mode, user should set maximal width of the arrows for the particular animation. The value of parameter (entitled as *darw*) responsible for maximal width of arrows can be set in the “Tool Bar” of visual map (in the “Options” window, “Combined Arrow” section, see detail description of the “Tool Bar” at the end of the section). If user chooses too low value for parameter *darw* the maximal width of the arrows is too small to distinguish changes in animated reaction rates. So, we recommend to choose the value of the parameter large enough.

Next step is to generate file of simulation data corresponding to the XML file with constructed visual map. To do it, choose Solver (ODE or Implicit) for generation of simulation data in “Get data from” window of “Options” section of DODE Visualization tabbed page. Then, run the model clicking “Save data for animation” and save them as PLT file.


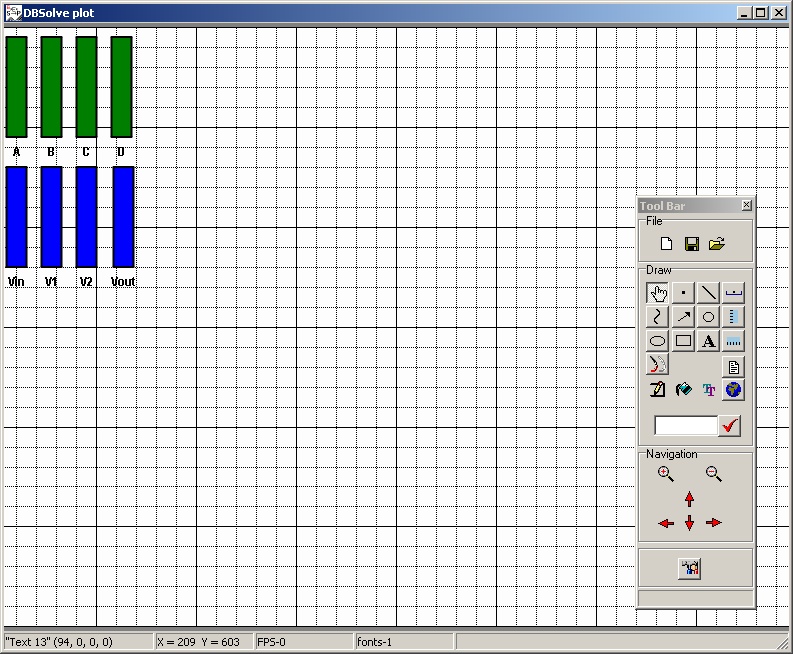


**Fig. A3.** Visual map for “Bar animation” mode.

When XML file with visual map and PLT file with simulation data corresponding to the visual map are generated, user can start with animation. To run Dynamic Visualization of the model calculation choose DBS Player (Start menu >> Programs >> DBSolve >> DBS Player). First open XML file with visual map by pressing “Open Scheme” button and then open the PLT data file by pressing “Open Data” button. Then, by clicking the "Play" button, one can view the Animation, and by clicking “Create AVI file” one can save the animation.

To look at and test the various animation modes without construction of the kinetic model, any modeler can use the set of preexisting models available at our web site (<http://biokinetics.ru/images/dbsolve/Examples_Visuzalization.zip>). Each preexisting model is accompanied with corresponding XML and PLT files. To run animation of these preexisting XML and PLT files, user should choose DBS Player (Start menu >> Programs >> DBSolve >> DBS Player). Then, open XML file with visual map (for example, *Scheme_TCA_Glutamate_ODE.xml)* from the folder “Examples” (Dynamic_Visualization_TCA_model\Arrows_visualization\) by pressing “Open Scheme” button. And then open the PLT data file with the same name (*Scheme_TCA_Glutamate_ODE.plt)* by pressing “Open Data”. Then, by clicking the "Play" button, one can view the Animation.

***Description of the “Tool Bar”***

Buttons from left to right: new model, save the current model, open a previously saved model.

Panel to select the shape and change its properties (fill, border color, font type for text).

Mode selection of objects.


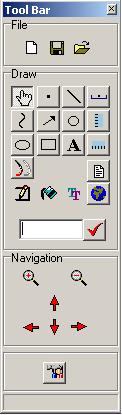


Panel for image scaling and positioning of the canvas.

Move the visual map

Insert a progress bar (horizontal or vertical).

Draw an

arrow

Insert a complicated arrow.

Draw an ellipse.

Search the text

Open the “OPTIONS” window

save to bmp

Insert a line of Beziers.


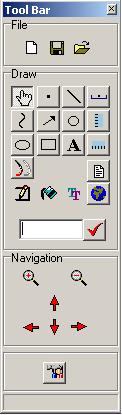


Write text

Draw a circle

Color and line thickness

Color and thickness of the figures

Customize text

Add a comment to an object (to select an object).

Add Internet-reference to the object (to select an object).

Draw a rectangle

# “OPTIONS” window


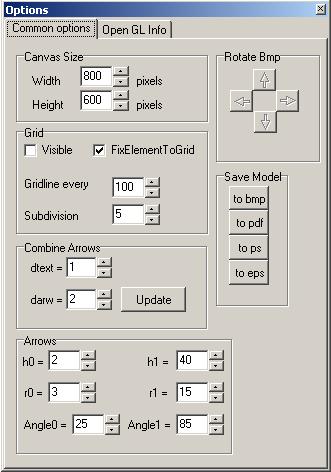


Change the size of the visual map

Set the grid of the visual map

Set combined arrows

Update changes

Maximal width of combine arrow

Indent arrows from the text.

Panel to edit form of the arrow-head

Link the shapes to the grid.


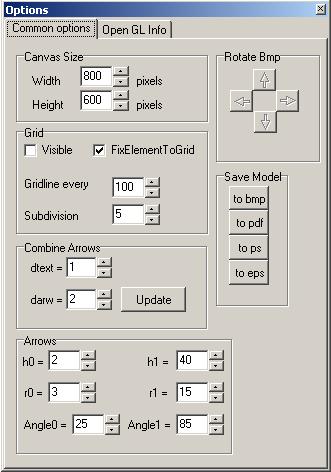


Turn bmp-images.

Save to formats: Bmp,

PostScript (PS),

Encapsulated PostScript (EPS) Portable Document Format (PDF).

B. Description of basic functionalities of DBSolve Optimum

DBSolve Optimum is a software package which enables the user to generate mathematical models of intermolecular processes of particular interest to biologists. These processes obviously include, but are not limited to, metabolic pathways, signal transduction and gene regulatory networks. DBsolve 7 is a successor of free available simulator DBsolve 5 [Goryanin, 1999]. Basic functionalities of DBSolve Optimum enable user

(1) to save and/or transfer kinetic models to other computer systems as an internal ASCII text files (with the suffix SLV), which contains all the necessary information or in SBML. DBSolve Optimum supports SBML format version 2 level 1 [Hucka, 2003],

(2) to construct the stoichiometry matrix and generate system of algebraic-differential equations taking into account conservation laws,

(3) to describe the dynamics of the model by means of measurable kinetic parameters,

(4) to solve “explicitly stated” formulae,

(5) to trace the changes in the steady state of the system as a result of variation of one or more of its parameters,

(6) to perform local bifurcation analysis,

(7) to collect raw experimental data,

(8) to fit the model against these data.

Significant changes and improvements have been made to meet current computational systems biology requirements. New features include:

- Integrated “C” compiler to speed up calculations. The feature is an essential for dealing with high dimensional whole cell models (i.e E.coli metabolic model ~1,000 equations);
- Improved user interface with syntax control in edit mode;
- User friendly inputs in different format;
- Simultaneous fitting of diverse experimental data (time series, dose response curves, etc.);
- Visualizer of fluxes/concentrations changes;
- Import/Export Microsoft Excel files;
- Import/Export Systems Biology Markup Language (SBML) files;
- 3D graphics;
- Bugs of previous version has been addressed;
- Possibility to use conditional operators such as: if (condition) {operators} else {operators}. This possibility allows to simulate piecewise continuous functions into the right hand sides of the differential equations, or algebraic equations.

Recently, DBSolve Optimum has been successfully employed for dynamic modeling and visualization of microbial central metabolism and gene regulation [Demin, 2008; Peskov, 2008a; Peskov, 2008b], signal transduction pathways [Moehren, 2002] and mitochondrial oxidative phosphorylation [Metelkin, 2006; Mogilevskaya, 2006] as well as to resolve problems arising in biomedicine [Goltsov, 2009; Smirnov, 2009] and biotechnology [Noble, 2006].

*RHS and IV files*

Since DBSolve Optimum is successor of software package DBSolve5 [Goryanin, 1999], representation of the model in this software packages is similar, i.e., ODE system and values of model parameters and variables are presented in the form of two plain-text files. Right hand sides of ODE system including reaction rates, conservation laws and explicit functions are defined in “RHS” file. Initial values of all variables of the model and values of all other parameters are defined in ”IV” file. Each line of the “IV” and “RHS” files has the following syntax: “variable = expression;”. Each line of the "RHS" and "IV" should be separated and ended with a comma point delimiter. In comparison to the previous version (DBSolve5) DBSolve Optimum allows to use conditional operators such as: if (condition) {operators} else {operators}. This possibility allows to simulate piecewise continuous functions into the right hand sides of the differential equations.

*Main window of DBSolve Optimum*

DBSolve Optimum interface (Fig. B1) consists of two windows: the basic window “DBSolve” and input/output window “Edit and View”. We will call basic window “DBSolve” as window ”Main” in future. The window ”Main” consists of the menu, two toolbars and the container of the tabsheets. Each tabsheet realizes specified functionality. Using the program menu it is possible to execute following operations: input/output, adjustment of the program to start the basic methods. The structure and purpose of each tabsheet is described below. In the first toolbar the buttons controlling most basic operations (open a model, save a model) and buttons controlling management of the view of the form ”Edit and View” (hide form, show form, form on the top of all) are collected. The second toolbar allows to start editing “IV”, “RHS” and “Comments” files, to start view “Messages” file - a window with warning messages and results of calculations as well as to stop calculation process pushing “Stop” button.


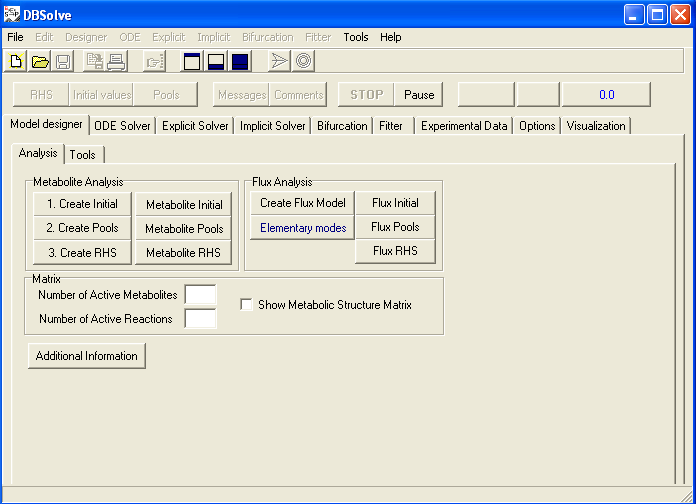


Fig. B1. Main window of DBSolve Optimum.

To simplify navigation through the texts placed in “RHS”, “IV” and “COMMENTS” windows “Tree view” mode has been developed. In framework of the mode the text of the files is divided by the sections. There are preset sections (such as “Pools”, “Rate Laws”, “Differential equations” in RHS file) and user defined sections. To define section the following syntax is used:

<? NB?> <? The section name?>

expressions etc. …

<? NE?> <? The section name?>

Each tabsheet of a window “Main” has corresponding tabsheet in ”Edit and View” window. Particularly, the model stoichiometric matrix is presented in a tabsheet “Metabolic Network”. Other tabsheets of ”Edit and View” window can be used to view plotted graphs resulted from calculation of the model using corresponding method of DBSolve Optimum. The graph component of the DBSolve Optimum package allows exporting charts to vector, raster formats, and MS Excel or plain text file. Below we consider in details functionality of each tabsheet of a window ”Main”. For example, various methods to create models are described in section ”Model Designer”.

*Model Designer*

Figure A1 shows the main application window with the “Model Designer” tabbed page in view. Pre-existing SLV files can be downloaded using the “Open” button. Upon selecting the file, the appropriate model parameters appear into the “Model Designer” page and grid of “Edit and View” page (the stoichiometric matrix). If starting from scratch, the user has to select one of the four alternatives from the “Model type” menu, which offers skeleton models for enzymes, metabolic pathways, receptor ligand interactions or some general process. Clicking the “New” button allows the creation of the SLV file into which all the subsequent details will be stored. A window then appears which allows selecting path and name where the new model should be stored. After it, program reports that skeleton model has been created successfully. Clicking the “OK” button user can begin to edit skeleton model. After it, user should define dimension of the system in the next fields: “Number of Reactions” and “Number of Metabolites” and fill stochiometric matrix of the system (see Table B1). On the top row of the grid, there are grey, numbered buttons which pressing results in popping up windows with details of the specified system component. Likewise, the grey buttons in the first column open a separate window that permits input of details of each reaction process, which include enzyme classification codes and sequence database identifiers (if appropriate) and the type of reaction mechanism. Each reaction name is unique as a sequence of alphabetic characters. The numbers on each row refer to the number of the components, defined by the column, participating in reaction. The model creation then finished by pressing sequentially next buttons: “Create Initials”, “Create Pools”, “Create RHS”. This results in creation of system of differential equation (RHS file) in accordance to stoichiometric matrix and initialization of model parameters with randomly selected numbers (IV file).

To identify these conservation laws, DBSolve Optimum reduces the stoichiometric matrix and sets up a vector of independent variables using systematic procedures for finding conservation laws from chemical [Bezdenenyh, 1973] and metabolic [Reder, 1988] kinetics.

*RCT format*

In DBSolve Optimum “RCT” format have been realized to simplify input of stoichiometric matrixes. The description of this format is given below. “RCT” file is ASCII text file with delimiters. Each string of this file describes stoichiometry of a reaction of the kinetic model and has a following syntax:

(reaction name1): (c1)*(substrate1) + (c2)*(substrate2) +... + (cn)*(substraten) = (d1)*(product1) + (d2)*(product2) +... + (dm)*(product m);

//you can write comments on a “C” language manner

/*

RCT file has multi line comment.

*/

Below you can find “RCT” file corresponding to kinetic model of Krebs cycle described in details in section B of Supplementary Materials:

AGC: Asp_in_ = Glu_in_ ; //aspartate-glutamate carrier

AspAT: Glu_in_ +OAA = Asp_in_ + KG_in_ ; //asparate aminotransferase

KGDH: KG_in_ + CoA = SucCoA;//α-ketoglutarate dehydrogenase

STK: SucCoA = Suc + CoA;// succinate thiokinase

SucDH: Suc = Fum;// succinate dehydrogenase

SucDHI: SDH + OAA = SDH-OAA;

/*

This reaction describes binding of oxaloacetate (OAA) to succinate dehydrogenase enzyme (SDH). This binding results in severe inhibition of the enzyme.

*/

FUM: Fum = Mal_in_;//fumarase

MDH: Mal_in_ = OAA;//malate dehydrogenase

KMC: KG_in_ = Mal_in_;// α-ketoglutarate-malate carrier

When downloaded and executed by DBSolve Optimum this “RCT” file is transformed to stoichiometric matrix of kinetic model of Krebs cycle presented in Table B1.

**Table B1.** Stoichiometric matrix of the kinetic model of Krebs cycle

|  | OAA | Glu_in_ | Asp_in_ | KG_in_ | CoA | SucCoA | Suc | SDH | SDH-OAA | Fum | Mal_in_ |
| --- | --- | --- | --- | --- | --- | --- | --- | --- | --- | --- | --- |
| AGC | 0 | 1 | -1 | 0 | 0 | 0 | 0 | 0 | 0 | 0 | 0 |
| AspAT | -1 | -1 | 1 | 1 | 0 | 0 | 0 | 0 | 0 | 0 | 0 |
| KGDH | 0 | 0 | 0 | -1 | -1 | 1 | 0 | 0 | 0 | 0 | 0 |
| STK | 0 | 0 | 0 | 0 | 1 | -1 | 1 | 0 | 0 | 0 | 0 |
| SucDH | 0 | 0 | 0 | 0 | 0 | 0 | -1 | 0 | 0 | 1 | 0 |
| SucDHI | -1 | 0 | 0 | 0 | 0 | 0 | 0 | -1 | 1 | 0 | 0 |
| FUM | 0 | 0 | 0 | 0 | 0 | 0 | 0 | 0 | 0 | -1 | 1 |
| MDH | 1 | 0 | 0 | 0 | 0 | 0 | 0 | 0 | 0 | 0 | -1 |
| KMC | 0 | 0 | 0 | -1 | 0 | 0 | 0 | 0 | 0 | 0 | 1 |

*ODE Solver*

To describe dynamics of the kinetic models DBSolve Optimum uses two algorithms: (1) an original implicit integration algorithm, with a step size control and with the Newton prediction-correction procedure for every integration step, similar to that of [Gear, 1984] and (2) a popular LSODE algorithm [Hindmarsh, 1983]. Both methods have special subroutines for getting output for user-defined time points, which is essential for fitting algorithms. To get the time evolution of the system, the user must go to the “ODE” page, define the variable to plot, time limit and accuracy, and press the “Solve” button. The results appear in the “Edit and View” window on the “ODE” tabsheet (see example of calculation of time dependence of NADH production by α-ketoglutarate dehydrogenase reaction *in vitro* in section C of Supplementary Materials).

*3D Plot*

In DBSolve Optimum 3D plot feature for “ODE solver” and “Implicit solver” has been realized. In framework of “ODE solver” this feature allows user to generate numerical data to plot 3D profile of any variable (indicated in the field “Y Axis” in the section “Plotting parameters”) on time and any model parameter (indicated in the field “Variable” in the section “Plot 3D”). In addition user should specify interval of variation of the parameter (indicated in the fields “Min and Max of selected variable or parameter”) and step of discrete mesh regarding the parameter (indicated in the field “Number of divisions”). As a result plain text file will be created. The file has three columns: values of time (first column), values of the parameter (second column) and values of variable. It is possible to use any program importing such files to plot a chart.

*Explicit Solver*

Users may have their own particular equation which they require solving and wish to be applied to a set of experimental data. DBSolve Optimum offers the facility to encode and solve such “explicitly stated” formulae. They should be typed at the bottom of the RHS window in the section “Explicit Function” and then solved by turning to the “Explicit Solver” tabbed page, where the appropriate variable, interval of variation of parameters and an initial step can be entered. Values of the chosen parameter vary on the selected interval, thus the initial step define amount of nodes on which “Explicit” function will be calculated. The method executed by pressing the “Solve” button. In section C of Supplementary Materials an example of calculation of Succinate thiokinase initial rate dependence on the concentration of its substrates and products is presented.

*Implicit Solver*

This method allows the user to trace the changes in the steady state of the system as a result of variation of one or more of its parameters. This procedure is very useful for determining any functional dependencies (such as overall steady-state flux, control coefficients, product concentration, some parameters of the model) against any external (substrate concentrations) or internal (enzyme concentrations) or some model parameter. It is especially useful in the case of non-linear algebraic systems which have no explicit solution or have multiple or unstable solutions. DBSolve Optimum includes a general continuation procedure, based on a tangent predictor continuation scheme [Khibnik et al., 1993]. A modified Newton corrector is employed which makes adaptive step sizes on the basis of estimates from the current tangent and secant vectors. This minimizes the possibility of jumping from one branch of a curve to another, and allows the user to optimize the next step size according to computed points on the curve. The “Implicit Solver” page contains three columns of parameter boxes: the first for the Solver parameters and the second and third for graphical display. 3D plot feature for “Implicit solver” has been realized. In section C of Supplementary Materials dependence of stationary glutamate consumption rate on concentration of external glutamate calculated from kinetic model of mitochondrial Krebs cycle is presented.

*Bifurcation Analysis*

Bifurcation theory is a more systematic and general theory of non-linear systems than the standard, steady-state analysis of metabolic networks. Computation of one or two-parameter bifurcation diagrams can quickly inform the user about what is possible for, or prohibited by, a particular type of non-linear model [Guckenheimer, 1983; Glass, 1988]. To calculate one and two-parameter diagrams of Equilibrium, Fold, Hopf, Flip and Focus-node bifurcations DBSolve Optimum uses numerical methods similar to LOCBIF [Khibnik et al., 1993]. All algorithms have been rewritten in “C ++” and modified to integrate with the DBSolve Optimum object-oriented environment. The Bifurcation Analyzer uses the same numerical continuation code as the Implicit Solver, but it is expanded with routines for the evaluation of bifurcation functions and calculating eigenvectors. Bifurcations have been found at points where black rectangles are drawn on the plot. Further details are put in the Message window.

*Experimental data*

Where pertinent data are available, DBSolve Optimum can use them for refining the model. The Experimental Data page contains a grid into which the data can be entered, either by typing or cutting-and-pasting from other sources. There are six columns per experiment and currently up to 53 experiments can be entered simultaneously. The first and second columns of each dataset (on the third row) contain the names of the model parameters that have been determined in the experiment, with the actual data supplied below. The third column contains weight of the experimental point of current curve; this value participates in the formula of discrepancy described in user guide. The fourth column contains the confidential interval for the experimental points. The fifth and sixth column of each dataset contains the name and value of any other parameter which was defined as part of the experiment. These extra details impose helpful constraints for fitting the model to the observed data. The first row of the first column contains the information on which method of calculation (implicit, explicit or ODE as described above) should be applied to the model for comparison with the observed data points. A 1 or 0 on the second row of the first column determines whether or not that particular dataset is to be included in the fitting. Pressing ”Plot” button and selecting the number of the experimental curve in experimental table will plot the output from the model (using the specified method) as a solid black line, against which the experimental data points are depicted as red squares (see Figures in section C of Supplementary Materials). In a “multiplot” mode the user can plot any set of experimental curves. Before run plotting, user should checkbox curves which are necessary plot in a ”Multiplot” form.

*Fitter*

This method can be used to fit a model to experimental data (thereby discovering the values with appropriate error margins of the models parameters under the conditions of the experiment). The tabsheet consists of three sub tabsheets: “Main”, “Options” and ”Advanced user”. Two last tabsheets contain information about parameters of the fitting.

Tabsheet “Main” allows user to set (1) linear and non linear limitations imposed to model parameters values; (2) the minimum value of the objective function which achievement stops the fitting; (3) intervals of variation of initial values. Linear constraints can be imposed by pressing the “Parameters” button and defining the initial, boundary and step values for specified parameters. In DBSolve Optimum feature to define nonlinear limitations on values of fitting parameters has been realized. Restrictions are defined in a “Fit conditions” window in a following manner:

F1(K1 … Kn)> F2(K1…Kn);

“Fitter” will search for values of parameters K1,…Kn allowing both minimizing objective function and holding true that specified function F1 depending on parameters K1, … Kn is more than function F2 depending on the same set of parameters.

To define intervals of a variation of initial values user should press to the button “IV fit” and enter names of variables and corresponding intervals of their variation in the table entitled ”Parameters for fitter”.

The ”Options” tabsheet allows user to select a fitting method and a method of calculation of the objective function. The fitting/optimization can exploit either a zero-order [Hooke, 1961] or first-order [Levenberg, 1944; Marquardt, 1963] algorithm. Fitting procedure often encounters difficulties caused by multiple minima, which may be a particular problem when many parameters are fitted. The “best” fit might not be easily found; however, to check the quality of the procedure, the standard deviation and confidence intervals for every active parameter as well as an ANOVA table are shown in the “Message window” to help users make their assessment. When fitting to experimental data, the objective residual function between theoretical and experimental points is calculated according to a least square or absolute value (modulus) formula. These are defined by the following equations:

F0 = ∑ (Yt_i_-Ye_i_) ^2^

F0 = ∑ |Yt_i_-Ye_i_ |

F0 = ∑ (Yt_i_-Ye_i_) 2/Ye_i_ ^2^

F0 = ∑ |Yt_i_-Ye_i_ | / | Ye_i_ |

where Yt_i_ and Ye_i_ are the theoretical and experimental values, respectively and F0 discrepancy. The “Absolute Fitting” box is checked if values of all datapoints are similar, otherwise relative fitting is applied.

The ”Advanced user” tabsheet allows user to set the maximum value of the time for achievement steady state for ODE as well as choose a mode of calculation of a standard deviation.

In section C of Supplementary Materials examples of fitting of experimental data by means of ODE Solver, Explicit Solver and Implicit Solver are presented.

*Options*

The “Options” tabsheet allows the user to adjust work of input/output system of the DBSolve Optimum. This tabsheet consists of three sub tabsheets: “Variables values”, “Plot”, “Report”. The “Variables values” tabsheet allows to define the parameters necessary for drawing “Family of Curves”: parameter name, its initial value, its step size and total number of steps. Also this tabsheet allows us to obtain initial value for any variable or parameter of the model. ”Plot” tabsheet allows us to control output of results of calculation to the plain text file and to chart. ”Report” tabsheet allows user to make reports on current model state.

**REFERENCES**

**Demin, O, Goryanin, I.** (2008) Kinetic Modelling in Systems Biology, Taylor & Francis (United States), pp.360

**Goryanin, I., Hodgman, C. and Selkov E.** (1999) Mathematical simulation and analysis of cellular metabolism and regulation. *Bioinformatics.* 15:749–758

**Hucka M. et al** (2003) The systems biology markup language (SBML): a medium for representation and exchange of biochemical network models. Bioinformatics. 1;19(4):524-31.

**Metelkin, Е, Goryanin, I, Demin, О.** (2006) Mathematical Modeling of Mitochondrial Adenine Nucleotide Translocase, Biophysical Journal, 15; 90 (2): 423-32.

**Mogilevskaya E., Demin O., Goryanin I.** (2006) Kinetic Model of Mitochondrial Krebs Cycle: Unravelling the Mechanism of Salicylate Hepatotoxic Effects. Journal of Biological Physics, pp. 245-271(27)

**Moehren, G., Markevic, N., Demin, O.V., Kiyatkin, A., Goryain, I., Kholodenko, B.N.** (2002) Temperature dependence of the epidermal growth factor receptor signaling network can be accounted for by a kinetic model. Biochemistry 41, 306-320

**Peskov K., Goryanin I., Demin O.** (2008a) Kinetic Model of Phosphofructokinase-1 from Escherichia coli. J Bioinform Comput Biol. 6(4), 843-67.

**Peskov K., Goryanin I., Prank K., Tobin F., Demin O.** (2008b) Kinetic Modeling of ace operon genetic regulation in Escherichia coli. J Bioinform Comput Biol 6(5): 933-959.

**Goltsov A., Maryashkin A., Swat M., Kosinsky Y., Humphery-Smith I., Demin O., Goryanin I., Lebedeva G.** Kinetic modelling of NSAID action on COX-1: focus on in vitro/in vivo aspects and drug combinations (2009) **Europ J Pharmac Sciences**, 36(1):122-36

**Smirnov S., Belashov A., Demin O.** Optimization of antimicrobial drug gramicidin S dosing regime using biosimulations (2008) **Europ J Pharmac Sciences,** 36(1):105-9

**Noble, M., Sinha, Y., Kolupaev, A., Demin, O., Earnshaw, D., Tobin, F., West, J., Martin, J.D., Qiu, C., Liu, W-S., DeWolf, W.E. Jr., Tew, D., Goryanin, I.** The kinetic model of the shikimate pathway as a tool to optimize enzyme assays for high-throughput screening. **Biotechnology and Bioengineering** (2006) 95, 560-571.

**Bezdeneznyh, A.** (1973) Inzenernye metody sostavlenija uravnenii skorostei reakzii i rascheta kineticheskih konstant. Chimija, Leningradskoe otdelenie.

**Reder,C.** (1988) Metabolic control theory: a structural approach. J. Theor. Biol., **135**, 175_201

**Gear,C.W. and Petzold,L.R.** (1984) ODE methods for the solution of differential algebraic systems. SIAM J. Num. Anal., **21**, 716_728Gear, 1984]

**Hindmarsh,A.C.** (1983) A systematized collection of ODE solvers. In Stepleman,R.S. et al. (eds), Scientific Computing. North Holland, Amsterdam, pp. 55_64. Also, http://www.codiciel.fr/netlib/odepack/doc

**Khibnik,A., Kuznetsov,Y., Levitin,V. and Nikolaev E.** (1993) Continuation techniques and interactive software for bifurcation analysis of ODEs and iterated maps. Physica D, **62**, 360_370.

**Guckenheimer,J. and Holmes,P.** (1983) Nonlinear Oscillations, Dynamical Systems, Bifurcations of Vector Fields. Springer-Verlag, Berlin.

**Glass,L. and Mackey,M.C.** (1988) From Clocks to Chaos: The Rhythms of Life. Princeton University Press, Princeton, NJ.

**Hooke,R. and Jeeves,T.A.** (1961) Direct search solution of numerical and statistical problems. J. Ass. Comput. Mach., **8**, 212_229.

**Levenberg,K.** (1944) A method of solution of certain nonlinear problems in least squares. Q. Appl. Math., **2**, 164_168.

**Marquardt,D.W.** (1963) An algorithm for least square estimation of non-linear parameters. SIAM J., **11**, 431_441.

C. Kinetic Model of mitochondrial Krebs cycle

Kinetic model of the citric acid cycle (also known as the tricarboxylic acid cycle, the TCA cycle, or the Krebs cycle) operating in mitochondria oxidizing glutamate and malate as substrates (see Fig. C1) has been developed in [Mogilevskaya, 2006]. The model represents following system of algebraic and differential equations:

$\frac{dGlu_{in}}{dt}=V_{AGC}-V_{AspAT}$;$\frac{dAsp_{in}}{dt}=V_{AspAT}-V_{AGC}$;$\frac{dOAA}{dt}=V_{MDH}-V_{AspAT}-V_{ISDH}$;$\frac{dKG_{in}}{dt}=V_{AspAT}-V_{KGDH}-V_{KMC}$;$\frac{dSucCoA}{dt}=V_{KGDH}-V_{STK}$;$\frac{dSuc}{dt}=V_{STK}-V_{SDH}$;$\frac{dFum}{dt}=V_{SDH}-V_{FUM}$;$\frac{dMal_{in}}{dt}=V_{FUM}-V_{MDH}+V_{KMC}$;$\frac{dCoA}{dt}=V_{STK}-V_{KGDH}$;$\frac{dSDH}{dt}=-V_{ISDH}$;$\frac{d(SDH-OAA)}{dt}=V_{ISDH}$

$$Glu_{in}+Asp_{in}=N_{tot}$$

$$CoA+SucCoA=CoA_{tot}$$

$$OAA+KG_{in}+SucCoA+Suc+Fum+Mal_{in}+SDH\_OAA=C_{tot}$$

$SDH+SDH\_OAA=SDH_{tot}$ (C1)

Here, N_tot_ (conservation of amino groups), CoA_tot_ (conservation of CoA), C_tot_ (conservation of four-carbon skeleton) and SDH_tot_ (conservation of succinate dehydrogenase).

Pool values and values of concentrations of metabolites are listed in Table C1.

**Fig. C1.** The scheme of the Krebs cycle oxidizing glutamate and malate as substrates. ***AGC****-aspartate-glutamate carrier;* ***AspAT****-asparate aminotransferase;* ***KGDH****- a-ketoglutarate dehydrogenase;* ***STK****-succinate thiokinase;* ***SDH****-succinate dehydrogenase;* ***FUM****-fumarase;* ***MDH****-malate dehydrogenase;* ***KMC****-a-ketoglutarate-malate carrier;* ***cI****-complex I;* ***SL****-salicyl-CoA ligase;* ***SGT*** *– salicyl-CoA-glycine acyltransferase.*

Description of individual enzymes of Krebs cycle.

*α-ketoglutarate dehydrogenase*

Rate equation of α-ketoglutarate dehydrogenase has been derived in [Mogilevskaya, 2006]:

$V_{KGDH}=\frac{KGDH\cdot(1+\frac{ADP}{K_{i}^{ADP}})\cdot k_{f}\frac{KG_{in}}{K_{m}^{KG_{in}}}\frac{CoA}{K_{m}^{CoA}}\frac{NAD}{K_{m}^{NAD}}}{\frac{CoA}{K_{m}^{CoA}}\frac{NAD}{K_{m}^{NAD}}(\frac{KG_{in}}{K_{m}^{KG_{in}}}+\frac{1+\frac{ATP}{K_{i}^{ATP}}}{1+\frac{Ca}{K_{i}^{Ca}}})+\frac{KG_{in}}{K_{m}^{KG_{in}}}(\frac{CoA}{K_{m}^{CoA}}+\frac{NAD}{K_{m}^{NAD}})(1+\frac{NADH}{K_{i}^{NADH}}+\frac{SucCoA}{K_{i}^{SucCoA}})}$(C2)

Here, KGDH is a concentration of α-ketoglutarate dehydrogenase; $K_{m}^{CoA},K_{m}^{NAD},K_{m}^{KG_{in}}$ are Michaelis constants for substrates; $K_{i}^{ADP},K_{i}^{ATP},K_{i}^{Ca},K_{i}^{NADH},K_{i}^{SucCoA}$ are inhibition constants for effectors; $k_{f}$ is catalytic constant. Parameters known from literature are KGDH, $k_{f}$, $K_{m}^{CoA}$, $K_{m}^{NAD}$, $K_{i}^{ADP}$,$K_{i}^{ATP},K_{i}^{Ca},K_{m}^{KG_{in}}$.

To estimate inhibition constants for NADH and SucCoA and make values of known parameters more precise experimental data published in [McCormack, 1979] have been used. Experiments have been designed as follows: reaction has been started by addition of $\alpha$-ketoglutarate dehydrogenase to the solution of substrates α−ketoglutarate, CoА and NAD and time dependences of NADH accumulation have been monitored with and without addition of effectors ADP and ATP. To quantitatively describe these experiments the following minimodel has been developed (Fig. C2):


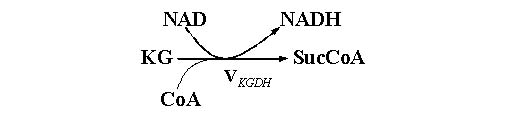
; $\frac{dCoA}{dt}=-V_{KGDH}$; $\frac{dNAD}{dt}=-V_{KGDH}$; $\frac{dNADH}{dt}=V_{KGDH}$; $\frac{dSucCoA}{dt}=V_{KGDH}$ (C3)

**Fig. C2.** The scheme of minimodel constructed to describe time dependences of NADH production catalyzed by α-ketoglutarate dehydrogenase.

Here, V_KGDH_ is rate equation for α-ketoglutarate dehydrogenase given by equation (C2); substrates and products of reaction catalyzed by α-ketoglutarate dehydrogenase are variables of the model (C3). Initial conditions for system of differential equations (C3) have been set in accordance with initial concentrations of substrates used in experiment [McCormack, 1979]: KG_in_=0.1 mM; NAD=1 mM; CoA=0.25 mM; NADH=0; SucCoA=0.

System of differential equation (C3) has four conservation laws:

$KG_{in}+SucCoA=C_{tot}^{KGDH}$ (conservation of four-carbon skeleton)

$CoA+SucCoA=CoA_{tot}^{KGDH}$ (conservation of CoA)

$KG_{in}+NADH=e_{tot}^{KGDH}$ (conservation of electrons)

$NADH+NAD=NAD_{tot}^{KGDH}$ (conservation of pyridine nucleotides)

Values of $NAD_{tot}^{KGDH}$, $e_{tot}^{KGDH}$, $CoA_{tot}^{KGDH}$, $C_{tot}^{KGDH}$ can be calculated from initial conditions. To estimate parameter values of equation (C2) we have fitted solution of system of differential equation (C3) to experimentally measured time dependencies of NADH accumulation. Fig. C3 demonstrates good fit of experimental data from [McCormack, 1979] (symbols) to theoretical curves generated by system of differential equations (C3). Values of kinetic parameters are listed in Table C2.


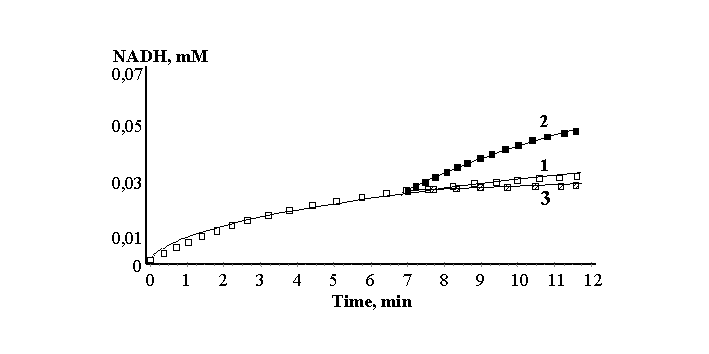


**Fig. C3.** Time dependence of NADH production by α-ketoglutarate dehydrogenase reaction presented by experimental points [McCormack, 1979] and described by the curves according to the system

(C3) with the following initial values (enzyme concentration was equal to 0,4 nM): 1) KG_in_=0.1 mM; NAD=1 mM; CoA=0.25 mM; NADH=0; SucCoA=0 (white squares);

2) 1.5 mM ADP was added on the 7^th^ minute (black squares); 3) 1.5 mM ATP was added on the 7^th^ minute (squares with oblique hatching)

*Succinate Thiokinase*

Rate equation of succinate thiokinase has been derived in [Mogilevskaya, 2006]:

$V_{STK}=\frac{STK\cdot\left(k_{1}k_{2}\frac{SucCoA}{K_{E-GDP-SucCoA}^{SucCoA}}\frac{GDP}{K_{E-GDP}^{GDP}}\frac{P}{K_{E-P}^{P}}-k_{-1}k_{-2}\frac{Suc}{K_{E-Suc}^{Suc}}\frac{GTP}{K_{E-GTP-CoA}^{GTP}}\frac{CoA}{K_{E-CoA}^{CoA}}\right)}{\begin{matrix} (k_{1}\frac{SucCoA}{K_{E-GDP-SucCoA}^{SucCoA}}\frac{GDP}{K_{E-GDP}^{GDP}}+k_{-2}\frac{GTP}{K_{E-GTP-CoA}^{GTP}}\frac{CoA}{K_{E-CoA}^{CoA}})(1+\frac{Suc}{K_{E-Suc}^{Suc}}+\frac{P}{K_{E-P}^{P}})+ \\ +(1+\frac{GDP}{K_{E-GDP}^{GDP}}+\frac{SucCoA}{K_{E-SucCoA}^{SucCoA}}+\frac{SucCoA}{K_{E-GDP-SucCoA}^{SucCoA}}\frac{GDP}{K_{E-GDP}^{GDP}}+\frac{GDP}{K_{E-GDP}^{GDP}}\frac{CoA}{K_{E-GDP-CoA}^{CoA}}+ \\ \frac{CoA}{K_{E-CoA}^{CoA}}+\frac{GTP}{K_{E-GTP}^{GTP}}+\frac{GTP}{K_{E-GTP-CoA}^{GTP}}\frac{CoA}{K_{E-CoA}^{CoA}}+\frac{SucCoA}{K_{E-GTP}^{GTP}}\frac{GTP}{K_{E-GTP-SucCoA}^{SucCoA}})(k_{2}\frac{P}{K_{E-P}^{P}}+k_{-1}\frac{Suc}{K_{E-Suc}^{Suc}}) \end{matrix}}$ (C4)

Here, STK is a concentration of Succinate Thiokinase, $k_{1},k_{-1},k_{2},k_{-2}$ are rate constants; $K_{E}^{S}$ is the dissociation constant for compound S from enzyme form E. Parameters known from literature are: $K_{m}^{CoA},K_{m}^{Suc},K_{m}^{SucCoA},K_{m}^{GDP},K_{m}^{GTP},K_{m}^{P}$ - Michaelis constants for CoA, Suc, SucCoA, GDP, GTP and P [Cha, 1964a]; catalytic constant k_f_ [Cha, 1964b]; and equilibrium constant K_eq_ [Kaufman, 1955]. Using the approach suggested in [Demin, 2004] we have expressed a number of parameters from equation (C4) in terms of kinetic parameters known from literature. Rate constants k_2_ and k_-1_ have been expressed from k_f_ and K_eq_ values:

$k_{2}=\frac{k_{f}k_{1}}{k_{1}-k_{f}}$; where $W=\frac{k_{f}^{3}K_{m}^{Suc}K_{m}^{GTP}K_{m}^{CoA}}{K_{eq}k_{1}k_{-2}^{2}K_{m}^{SucCoA}K_{m}^{GDP}K_{m}^{P}}$.

Six dissociation constants have been expressed through Michaelis constants:

$K_{E-GDP-SucCoA}^{SucCoA}=K_{m}^{SucCoA}\frac{k_{1}+k_{2}}{k_{2}}$; $K_{E}^{GDP}=K_{m}^{GDP}\frac{k_{1}+k_{2}}{k_{2}}$; $K_{E-GDP-CoA-P}^{P}=K_{m}^{P}\frac{k_{1}+k_{2}}{k_{2}}$; $K_{E}^{Suc}=K_{m}^{Suc}\frac{k_{-1}+k_{-2}}{k_{-2}}$; $K_{E-GTP-CoA}^{GTP}=K_{m}^{GTP}\frac{k_{-1}+k_{-2}}{k_{-2}}$; $K_{E}^{CoA}=K_{m}^{CoA}\frac{k_{-1}+k_{-2}}{k_{-2}}$.

Taking into account these relationships we have decreased the number of unknown parameters of equation (C4) from 14 to 6. Remained undetermined parameters were: k_1_, k_-2_, $K_{E-SucCoA}^{SucCoA}$, $K_{E-GDP-CoA}^{CoA}$, $K_{E-GTP}^{GTP}$, $K_{E-GTP-SucCoA}^{SucCoA}$. We have estimated their values from experimental data [Cha, 1964a] where dependences of the initial rate of succinate thiokinase on substrates and products have been measured. Moreover, the fitting of the rate equation (C4) to experimental data has allowed us to identify Michaelis constants for substrates and products more precisely (see Table C2). Fig. C4 demonstrates that experimental data from [Cha, 1964a] (symbols) and theoretical curves generated by equation (C4) closely coincide.

**a) b)**

**
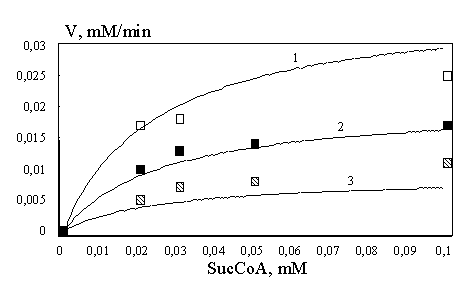

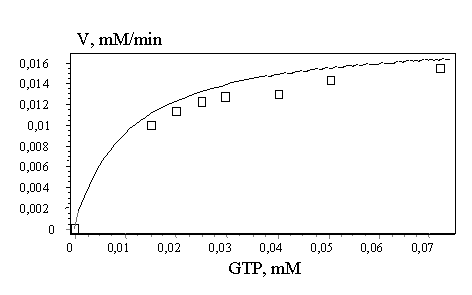
**

**Fig. C4.** Succinate thiokinase initial rate dependence on the concentration of substrates and products presented by experimental points [Cha, 1964a] and described by the curves according to the rate equation

(C4) in the following conditions (enzyme concentration was defined to be 0.05 μM):

a) GDP=0,05 mM; SucCoA, mM: 1) - 0.05 (white squares); 2) - 0.03 (black squares); 3) - 0.02 (squares with oblique hatching);

b) Suc=1 mM; CoA=0.1 mM (white squares)

*Aspartate-glutamate carrier*

Rate equation of aspartate-glutamate carrier has been derived in [Mogilevskaya, 2006]:

$$V_{AGC}=AGC\cdot\frac{k_{1}k_{2}\frac{Glu_{out}}{K_{m}^{Glu_{out}}}\frac{Asp_{in}}{K_{m}^{Asp_{in}}}\frac{H_{out}}{K_{m}^{H_{out}}}-k_{-1}k_{-2}\frac{Glu_{in}}{K_{m}^{Glu_{in}}}\frac{Asp_{out}}{K_{m}^{Asp_{out}}}\frac{H_{in}}{K_{m}^{H_{in}}}}{\begin{matrix} (k_{1}\frac{Glu_{out}}{K_{m}^{Glu_{out}}}\frac{Asp_{in}}{K_{m}^{Asp_{in}}}\frac{H_{out}}{K_{m}^{H_{out}}}+k_{-2})(1+\frac{H_{in}}{K_{m}^{H_{in}}})\cdot(1+\frac{Glu_{in}}{K_{m}^{Glu_{in}}})(1+\frac{Asp_{out}}{K_{m}^{Asp_{out}}})+(k_{1}\frac{Glu_{in}}{K_{m}^{Glu_{in}}}\frac{Asp_{out}}{K_{m}^{Asp_{out}}}\frac{H_{in}}{K_{m}^{H_{in}}}+ \\ +k_{2})(1+\frac{H_{out}}{K_{m}^{H_{out}}})\cdot(1+\frac{Glu_{out}}{K_{m}^{Glu_{out}}})(1+\frac{Asp_{in}}{K_{m}^{Asp_{in}}}) \end{matrix}}$$

Here, AGC is the concentration of the aspartate-glutamate carrier, $K_{m}^{Glu_{out}},K_{m}^{Asp_{in}},K_{m}^{H_{out}},K_{m}^{Glu_{in}},K_{m}^{Asp_{out}},K_{m}^{H_{in}}$are the Michaelis constants for intra- and extramitochondrial glutamate, aspartate and proton. These parameters depend on electric potential:

$K_{m}^{H_{in}}=K_{m,0}^{H_{in}}*e^{\delta_{1}\frac{\Delta\psi}{RT/F}}$; $K_{m}^{H_{out}}=K_{m,0}^{H_{out}}*e^{-\delta_{3}\frac{\Delta\psi}{RT/F}}$; $k_{1}=k_{1}^{0}*e^{(1-\alpha)\delta_{2}\frac{\Delta\psi}{RT/F}}$; $k_{-1}=k_{-1}^{0}*e^{-\alpha\delta_{2}\frac{\Delta\psi}{RT/F}}$

where $\Delta\psi>0$ is a transmembrane potential; Т is the absolute temperature; R is the universal gas constant; F is the Faraday’s constant; $\delta_{i}$is a part of the potential, consumed by *i-*th stage; $\alpha$ is a part of the potential that influences reverse reaction. We have assumed that δ_1_=0.1, δ_2_=0.8, δ_3_=0.1, $\alpha$=0.5. Values of other parameters are listed in Table B2.

*Aspartate aminotransferase*

Rate equation of aspartate aminotransferase has been derived in [Mogilevskaya, 2006]:

$V_{AspAT}=AspAT*\frac{k_{f}^{2}\frac{OAA}{K_{m}^{OAA}}\frac{Glu_{in}}{K_{m}^{Glu_{in}}}-k_{r}^{2}\frac{Asp_{in}}{K_{m}^{Asp_{in}}}\frac{KG_{in}}{K_{m}^{KG_{in}}}}{\begin{matrix} (k_{f}\frac{Glu_{in}}{K_{m}^{Glu_{in}}}+k_{r}\frac{Asp_{in}}{K_{m}^{Asp_{in}}})(1+\frac{k_{r}}{k_{-1}}\frac{KG_{in}}{K_{m}^{KG_{in}}}+\frac{k_{1}-k_{f}}{k_{1}}\frac{OAA}{K_{m}^{OAA}})+ \\ +(k_{r}\frac{KG_{in}}{K_{m}^{KG_{in}}}+k_{f}\frac{OAA}{K_{m}^{OAA}})*(1+\frac{k_{f}}{k_{1}}\frac{Glu_{in}}{K_{m}^{Glu_{in}}}+\frac{k_{-1}-k_{r}}{k_{-1}}\frac{Asp_{in}}{K_{m}^{Asp_{in}}}) \end{matrix}}$ (C4)

Here, AspAT is concentration of aspartate aminotransferase; $K_{m}^{OAA},K_{m}^{Asp_{in}},K_{m}^{Glu_{in}},K_{m}^{KG_{in}}$ are Michaelis constants for substrates and products; $k_{f},k_{r}$ are turnover numbers for forward and reverse directions, $k_{1},k_{-1}$ are rate constants of individual reaction steps. All parameter values are listed in Table C2.

*Succinate Dehydrogenase (SDH)*

Rate equation of succinate dehydrogenase carrier has been derived in [Mogilevskaya et al, JBP]:

$V_{SDH}=SDH\frac{k_{f}\frac{Suc}{K_{E-Suc}^{Suc}}\frac{Q}{K_{m}^{Q}}-k_{r}\frac{Fum}{K_{E-Fum}^{Fum}}\frac{QH_{2}}{K_{m}^{QH_{2}}}}{1+\frac{Suc}{K_{E-Suc}^{Suc}}+\frac{Q}{K_{m}^{Q}}\frac{K_{m}^{Suc}}{K_{E-Suc}^{Suc}}+\frac{Suc}{K_{E-Suc}^{Suc}}\frac{Q}{K_{m}^{Q}}+\frac{Fum}{K_{,E-Fum}^{Fum}}+\frac{QH_{2}}{K_{m}^{QH_{2}}}\frac{K_{m}^{Fum}}{K_{E-Fum}^{Fum}}+\frac{Fum}{K_{E-Fum}^{Fum}}\frac{QH_{2}}{K_{m}^{QH_{2}}}}$ (C5)

Here, SDH is a concentration of Succinate Dehydrogenase; $k_{f},k_{r}$ are turnover numbers in forward and reverse directions; $K_{E}^{S}$ is the dissociation constants for compound S from enzyme form E; $K_{m}^{Q},K_{m}^{QH2},K_{m}^{Suc},K_{m}^{Fum}$ are Michaelis constants for ubiquinone, ubiquinol, succinate and fumarate. The values of all parameters of equation (C5) are listed in Table C2.

*The process of SDH inactivation*

Rate equation of SDH inactivation has been derived in [Mogilevskaya, 2006]:

$V_{ISDH}=k_{i}\cdot SDH\cdot OAA-k_{-i}\cdot(SDH-OAA)$ (C6)

The values of all parameters of equation (C6) are listed in Table C2.

*Fumarase*

Rate equation of fumarase has been derived in [Mogilevskaya, 2006]:

$V_{FUM}=FUM\frac{k_{f}\frac{Fum}{K_{m}^{Fum}}-k_{r}\frac{Mal_{in}}{K_{m}^{Mal_{in}}}}{1+\frac{Fum}{K_{m}^{Fum}}+\frac{Mal_{in}}{K_{m}^{Mal_{in}}}}$ (C7)

The values of all parameters of equation (C7) are listed in Table C2.

*Malate dehydrogenase*

Rate equation of malate dehydrogenase has been derived in [Mogilevskaya, 2006]:

$V_{MDH}=\frac{MDH\cdot k_{f}k_{r}(Mal_{in}\cdot NAD/K_{eq}-NADH\cdot OAA)}{\begin{matrix} K_{i}^{NADH}K_{m}^{OAA}k_{r}+K_{m}^{OAA}k_{r}\cdot NADH+K_{m}^{NADH}k_{r}\cdot OAA+k_{r}\cdot NADH\cdot OAA+ \\ +K_{m}^{NAD}k_{f}\cdot Mal_{in}/K_{eq}+K_{m}^{Mal}k_{f}\cdot NAD/K_{eq}+k_{f}\cdot Mal_{in}\cdot NAD/K_{eq}+ \\ +K_{m}^{NAD}k_{f}\cdot NADH\cdot Mal_{in}/K_{i}^{NADH}K_{eq}+K_{m}^{NADH}k_{r}\cdot OAA\cdot NAD/K_{i}^{NAD}+ \\ +k_{r}\cdot NADH\cdot OAA\cdot Mal_{in}/K_{i}^{Mal}+k_{f}\cdot OAA\cdot Mal_{in}\cdot NAD/K_{i}^{OAA}K_{eq} \end{matrix}}$ (C8)

The values of all parameters of equation (C8) are listed in Table C2.

*α−Ketoglutarate-Malate Carrier (KMC)*

Rate equation of α−ketoglutarate-malate carrier has been derived in [Mogilevskaya, 2006]:

$V_{KMC}=KMC\frac{k_{f}^{2}\frac{k_{2}}{k_{1}}\frac{KG_{in}}{K_{m}^{KG_{in}}}\frac{Mal_{out}}{K_{m}^{Mal_{out}}}-k_{r}^{2}\frac{k_{-2}}{k_{-1}}\frac{KG_{out}}{K_{m}^{KG_{utt}}}\frac{Mal_{in}}{K_{m}^{Mal_{in}}}}{\begin{matrix} (\frac{k_{f}^{2}}{k_{1}}\frac{KG_{in}}{K_{m}^{KG_{in}}}\frac{Mal_{out}}{K_{m}^{Mal_{out}}}+k_{-2})(1+\frac{k_{r}}{k_{-1}}\frac{KG_{out}}{K_{m}^{KG_{utt}}}(1+\frac{KG_{out}}{K_{i}^{KG_{out}}}))(1+\frac{k_{r}}{k_{-1}}\frac{Mal_{in}}{K_{m}^{Mal_{in}}})+ \\ +(k_{2}+\frac{k_{r}^{2}}{k_{-1}}\frac{KG_{out}}{K_{m}^{KG_{utt}}}(1+\frac{KG_{out}}{K_{i}^{KG_{out}}})\frac{Mal_{in}}{K_{m}^{Mal_{in}}})(1+\frac{k_{f}}{k_{1}}\frac{KG_{in}}{K_{m}^{KG_{in}}})(1+\frac{k_{f}}{k_{1}}\frac{Mal_{out}}{K_{m}^{Mal_{out}}}) \end{matrix}}$ (C9)

The values of all parameters of equation (C9) are listed in Table C2.

Verification of kinetic model of mitochondrial Krebs cycle against experimental data measured at steady state.

To estimate values of the unknown parameters, we have fitted the system of algebraic - differential equations (C1) against the dependence of steady-state flux of glutamate consumption on concentration of glutamate in medium. Fig. C5 demonstrates that experimental data from [23] (symbols) and the theoretical curve generated by the system of algebraic-differential equations (C1) closely coincide. Values of intramitochondrial enzyme concentrations and kinetic parameters obtained are listed in Table C2 (all parameters obtained by fitting to experimental data [La Noue, 1979] are marked by asterisk).


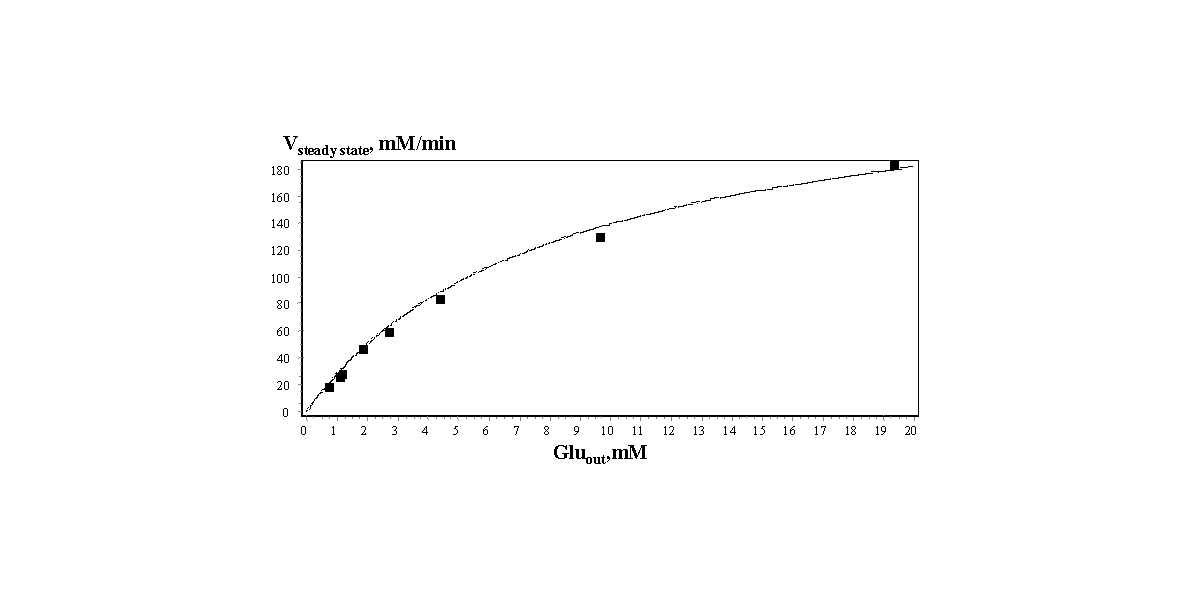


**Fig. C5.** Dependence of stationary glutamate consumption rate by mitochondrial suspension on glutamate concentration. Simulation results and experimental points from [La Noue, 1979] under the following conditions: Glu_out_=0 - 20 mM, Mal_out_=3,7 mM, pH_out_ =7.4.

References

Cha S., Parks R.E. Succinic thiokinase.II. Kinetic studies: initial velocity, product inhibition, and effect of arsenate. 1964a. J. Biol. Chem. 239, 1968-1977.

Cha S., Parks R.E. Succinic thiokinase.I.Purification of the enzyme from pig heart. 1964b. J. Biol. Chem. 239, 1961-1967.

Demin OV, I.I. Goryanin, S.Dronov, G.V.Lebedeva . Kinetic model of imidazole glycerol phosphate synthetase of Escherichia coli. 2004. Biokhimiya. 69, 1625-1638.

Dierks T, Kramer R. Asymmetric orientation of the reconstituted aspartate/glutamate carrier from mitochondria. 1988. Biochim. Biophys. Acta. 937, 112-126.

Hamada M, Koike K, Nakaula Y, Hiraoka T, Koike M, Hashimoto T. A kinetic study of the α-keto acid dehydrogenase complexes from pig heart mitochondria. 1975. J. Biochem. 77, 1047-1056.

Heyde E, Ainsworth S. Kinetic Studies on the Mechanism of the Malate Dehydrogenase

Reaction. 1968. J. Biol. Chem. 243, 2413-2423.

Garber AJ, Hanson RW. The interrelationships of the various pathways forming gluconeogenic

precursors in guinea pig liver mitochondria. 1971. J. Biol. Chem. 246, 589-598.

Grivennikova VG, Gavrikova EV, Timoshin AA, Vinogradov AD. Fumarate reductase activity of bovine heart succinate-ubiquinone reductase. New assay system and overall properties of the reaction. 1993. Biochim. Biophys. Acta. 1140, 282-292.

Fahien LA, Teller JK. Glutamate-malate metabolism in liver mitochondria. A model constructed on the basis of mitochondrial levels of enzymes, specificity, dissociation constants, and stoichiometry of hetero-enzyme complexes. 1992. J. Biol. Chem. 267, 10411-10422.

Indiveri C., Dierks T., Kramer R., Palmieri F. Reaction mechanism of the reconstituted

oxoglutarate carrier from bovine heart mitochondria. 1991. Eur. J. Biochem. 198, 339-347.

Kaplan E.H., Kennedy J., Davis J. Effects of salicylate and other benzoates on oxidative

enzymes of the tricarboxylic acid cycle in rat tissue homogenates. 1954. Archives of

Biochemistry. 51, 47-61.

Kaufman S., Alivisatos S.G.A. Purification and properties of the phosphorilating enzyme from spinach. 1955. J. Biol. Chem. 216, 141-152.

Kotlyar AB, Vinogradov AD. Dissociation constants of the succinate dehydrogenase complexes with succinate, fumarate and malonate. 1984. Biokhimiya. 49, 511-518.

Kuramitsu S, Inoue K, Kondo K, Aki K, Kagamiyama H. Aspartate aminotransferase isozymes from rabbit liver. Purification and properties. 1985. J. Biochem. 97, 1337-1345.

La Noue KF, Duszynski J, Watts JA, McKee E. Kinetic properties of aspartate transport in rat

heart mitochondrial inner membranes. 1979. Archives of Biochemistry and Biophysics. 195,

578-590.

Massey V. The composition of the α-ketoglutarate dehydrogenase complex. 1960. Biochim. Biophys. Acta. 38, 447-460.

McCormack JG, Denton RM. The effects of calcium ions and adenine nucleotides on the activity of pig heart 2-oxoglutarate dehydrogenase complex. 1979. Biochem. J. 180, 533-544.

Mogilevskaya E., Demin O., Goryanin I. Kinetic Model of Mitochondrial Krebs Cycle: Unraveling the Mechanism of Salicylate Hepatotoxic Effects. Journal of Biological Physics, 2006, vol.32(3-4), pp.245-271.

Siess EA, Kientsch-Engel RI, Wieland OH. Concentration of free oxaloacetate in the

mitochondrial compartment of isolated liver cells. 1984. Biochem. J. 218, 171-176.

Vinogradov AD. Succinate-ubiquinone reductase of the respiratory chain. 1986. Biokhimiya. 51,

1944-1973.

**Table C1.**Krebs cycle metabolite concentrations.

| Metabolite | Concentration, mM | Metabolite | Concentration, mM |
| --- | --- | --- | --- |
| KG_in_ | 0.018 | Suc | 0.007 |
| KG_out_ | 0.54 | Asp_out_ | 0 |
| Glu_in_ | 7.3 | Fum | 1.94 |
| Glu_out_ | 20 | NAD | 2 |
| OAA | 0.0002 | NADH | 1 |
| Mal_in_ | 1.16 | ATP+ADP | 12 |
| Mal_out_ | 0 | ATP/ADP | 9.4 |
| Asp_in_ | 0.3 | P | 5 |
| SucCoA | 0.63 | GDP | 0.2 |
| CoA | 0.37 | GTP | 1.8 |
| N_tot_ | 7,6 | Q | 19 |
| SDH_tot_ | 0,05 | QH_2_ | 1 |
| Ca^2+^ | 0.001 | H_in_ | 5.2e-6 |
| Δψ | 139 mV | H_out_ | 3.98e-5 |
| C_tot_ | 3.801 | Gly | 1 |
| CoA_tot_ | 1 | SDH-OAA | 0.0458 |
|  |  | SDH | 0.0042 |

**Table C2.** Kinetic parameters values of the Krebs cycle enzymes known from literature and estimated via fitting of rate equations to literature experimental data (Michaelis constants, dissociation constants and enzymes concentrations are in mM, rate constants are in 1/min).

| Enzyme designation | Literature values of kinetic parameters taken from (Ref.) | Kinetic parameters values estimated via fitting to experimental data taken from (Ref.) |
| --- | --- | --- |
| AGC | $K_{m}^{Glu_{out}}$=0.25; $K_{m}^{Glu_{in}}$=3; $K_{m}^{Asp_{out}}$=0.12 [Dierks, 1988]  $K_{m}^{Asp_{in}}$=0.0435 [La Noue, 1979] | *AGC=2; k_1,0_=99800; k_-1,0_=9940; k_2_=100000  k_-2_=9940; $K_{m}^{H_{in}}$=0.00004; $K_{m}^{H_{out}}$=0.01; *$K_{m}^{H_{out}}$=0.1; $K_{m}^{Glu_{out}}$*=9.3; [La Noue, 1979] |
| AspAT | AspAT=0.14 [Fahien, 1992]; k_r_=51870; $K_{m}^{KG_{in}}$=6.9; $K_{m}^{Asp_{in}}$=1.9 [Kuramitsu, 1985]; $K_{m}^{OAA}$=0.088 [Garber, 1971]; Keq=6.6 [Siess, 1984] | *AspAT=1.5; k_1_=5e7; k_f_=10000 [La Noue, 1979]; $K_{m}^{Glu_{in}}$=0.55; k_-1_=51999 [Kuramitsu, 1985] |
| KGDH | KGDH=0.002 [Fahien, 1992]; k_f_=83110 [Massey, 1960]  $K_{m}^{CoA}$=0.0027 [40]; $K_{m}^{NAD}$=0.05 [Hamada, 1975]; $K_{m}^{KG_{in}}$=0.2; $K_{i}^{ATP}$ =0.1; $K_{i}^{ADP}$ =0.1; $K_{i}^{Ca}$ =0.0012 [McCormack, 1979] | *KGDH=1; *$K_{i}^{ADP}$=0.005 [La Noue, 1979]  $K_{m}^{KG_{in}}$=0.03; $K_{m}^{CoA}$=0.002; $K_{m}^{NAD}$=0.93; $K_{i}^{SucCoA}$=0.011; $K_{i}^{NADH}$ =0.0018; $K_{i}^{ATP}$=0.01;$K_{i}^{ADP}$=0.56[McCormack, 1979];$K_{i}^{Sal}$=0.001[Kaplan, 1954] |
| STK | $K_{m}^{Suc}$=0.4-0.8 $K_{m}^{CoA}$=0.005-0.02; $K_{m}^{SucCoA}$=0.01-0.06; $K_{m}^{GDP}$=0.002-0.008; $K_{m}^{GTP}$=0.05-0.01; $K_{m}^{P}$=0.2-0.7 [Cha, 1964a]; k_f_=10780 [Cha, 1964b]; K_eq_=3.7 [Kaufman, 1955] | $K_{m}^{Suc}$=0.81; $K_{m}^{CoA}$=0.017; $K_{m}^{SucCoA}$=0.024  $K_{m}^{GDP}$=0.007; $K_{m}^{GTP}$=0.000068; $K_{m}^{P}$=1.5  k_1_=1700000; k_-1_=1149; k_2_=10000;  k_-2_=1990000; $K_{d}^{SucCoA}$=0.029; $K_{d,E-GDP-CoA}^{CoA}$=0.00038; $K_{d}^{GDP}$=0.14; $K_{d,E-GTP-SucCoA}^{SucCoA}$=0.49 [Cha, 1964a]; *STK=1 [La Noue, 1979] |
| SDH | SDH=0.05; k_f_=10000; k_r_=102 [Vinogradov, 1986]  $K_{m}^{Suc}$=0.13; $K_{m}^{Q}$=0.0003; $K_{m}^{QH_{2}}$=0.0015; $K_{m}^{Fum}$=0.025 [Grivennikova, 1993]  $K_{d,E-Suc}^{Suc}$=0.01;$K_{d,E-Fum}^{Fum}$=0.29 [Kotlyar, 1984] | $K_{m}^{Suc}$=0.084; $K_{d,E-Suc}^{Suc}$=0.29 [Kotlyar, 1984]  k_f_=1e6[La Noue, 1979]  $K_{i}^{Sal}$=7e-5 [Kaplan, 1954] |
| FUM | FUM=2.27e-4; $K_{m}^{Fum}$=0.047; $K_{m}^{Mal_{in}}$=0.017; k_f_=90721; k_r_=71342 [54] | *FUM=0.5; *$K_{m}^{Fum}$=0.01 [La Noue, 1979]  $K_{m}^{Fum}$=0.036; k_f_=90722 [55] |
| MDH | k_f_=5.4e5; k_r_=8.6e3; MDH=9.03e-4; $K_{m}^{OAA}$=0.0795; $K_{m}^{Mal_{in}}$=0.386; $K_{m}^{NAD}$=0.0599; $K_{m}^{NADH}$=0.26; $K_{i}^{OAA}$=0.0055; $K_{i}^{Mal_{in}}$=0.36  $K_{i}^{NAD}$=1.1; $K_{i}^{NADH}$=0.0136 ; K_eq_=8000; [Heyde, 1968] | *MDH=1 [La Noue, 1979] |
| KMC | k_f_=325; k_r_=309; $K_{m}^{Mal_{out}}$= 1.36; $K_{m}^{Mal_{in}}$=0.71; $K_{m}^{KG_{in}}$=0.17; $K_{m}^{KG_{out}}$=0.31; K_eq_=1 [Indiveri, 1991] | *KMC=2 [La Noue, 1979]  k_1_=858; $K_{i}^{KG_{out}}$=4.2e-3 [Indiveri, 1991] |
| Oxaloacetate binding to SDH (ISDH) | k_i_=1200 1/min*mM; k_-i_=0.02 1/min [Vinogradov, 1986] |  |

* Parameters values estimated from verification of the whole model
